# Natural products modulate programmed cell death signaling mechanism for treating endometriosis: a review

**DOI:** 10.3389/fphar.2026.1742212

**Published:** 2026-01-29

**Authors:** Zhen Zhao, Fangyuan Liu, Yang Yu, Ying Shen, Danni Ding, Fengjuan Han

**Affiliations:** 1 First Clinical Medical College, Heilongjiang University of Chinese Medicine, Harbin, China; 2 The First Affiliated Hospital of Heilongjiang University of Chinese Medicine, Harbin, China

**Keywords:** EMS, mechanism, natural products, PCD, signaling pathways, therapy

## Abstract

Endometriosis (EMs) is a gynecological inflammatory disease that depends on estrogen. Its chief symptoms include dysmenorrhea, chronic pelvic pain, reduced fertility, and pelvic masses. Although various hormonal therapies and surgical treatments are available, their long-term effectiveness is limited, recurrence rates are high, and side effects are significant. Programmed cell death (PCD) is a genetically regulated mechanism of cell clearance that includes apoptosis, autophagy, ferroptosis, pyroptosis, and necroptosis. Numerous studies showed that dysregulation of PCD is strongly associated with the development of EMs, suggesting that targeting key molecular mechanisms of PCD could be a promising therapeutic strategy. Natural products, known for their multitarget activity and low toxicity, show unique advantages in modulating PCD in EMs. This review elucidates the regulatory mechanisms of various PCD pathways in EMs and their interactions with key signaling cascades, including PI3K/Akt/mTOR, MAPK, NF-κB, and Bcl-2. Furthermore, it explores how natural products modulate these PCD mechanisms and related pathways, providing insights into their therapeutic potential at the molecular level. We used “endometriosis,” “programmed cell death,” “natural products”, and “signaling pathway” as keywords to systematically search the PubMed, Web of Science, and CNKI databases for relevant literature published in the past 10 years. A total of 55 studies were included, highlighting recent advances in regulating EMs progression through PCD modulation by natural products. The goal of this review is to provide a theoretical foundation for improving current treatments for EMs and to offer practical recommendations for future research.

## 1 Introduction

Endometriosis (EMs) is a gynecological inflammatory disease that depends on estrogen. This leads to a range of clinical sequelae, including chronic pelvic pain, infertility, and pelvic mass formation. It affects about 5%–10% of women (Taylor et al., 2021). Current evidence suggests that EMs is closely associated with chronic inflammation, angiogenesis, and dysregulation of the immune microenvironment (Kapoor et al., 2021). Current first-line treatments are hormonal therapy and surgical intervention. While these can temporarily relieve symptoms, they often cause adverse effects and are prone to recurrence. Therefore, it is urgent to develop new therapeutic strategies that are effective and have fewer side effects.

**FIGURE 1:**
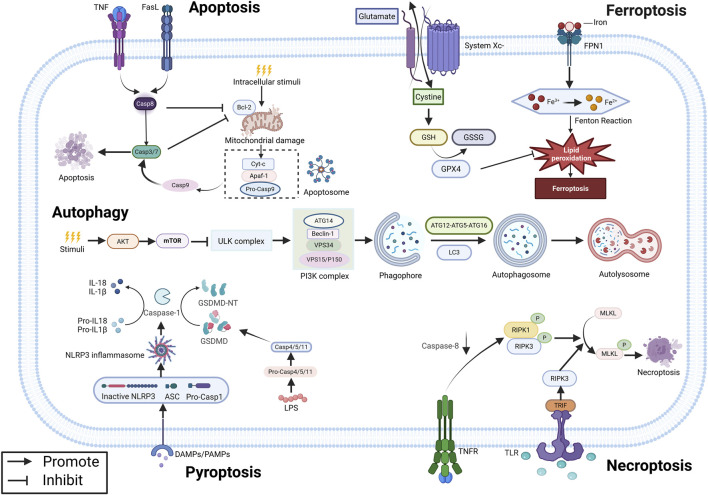
Mechanism of PCD in EMs. Created with Biorender.com.

**FIGURE 2:**
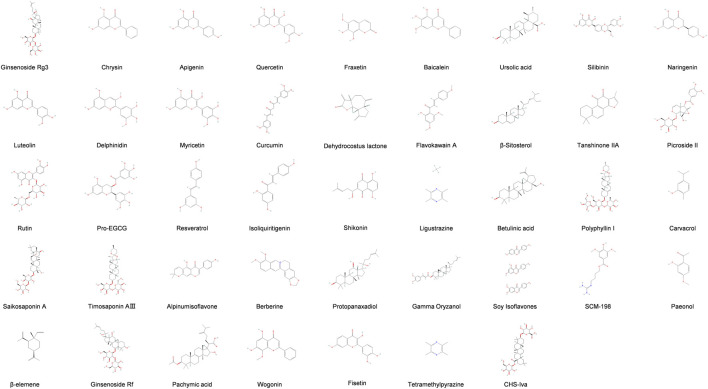
Chemical structures of natural products.

Programmed cell death (PCD) is an active cellular clearance process that includes apoptosis, autophagy, ferroptosis, pyroptosis, and necroptosis (Figure 1). It plays an essential role in maintaining physiological balance, controlling inflammation, and preserving immune stability (Bedoui et al., 2020). Dysregulation of PCD is implicated in the pathogenesis of EMs, positioning targeting PCD as a promising therapeutic target (Huang E. et al., 2024). Recently, natural products have attracted attention due to their multi-target mechanisms, good safety, and therapeutic effects on various pathologies, including cancer and inflammatory diseases (Vahdat-Lasemi et al., 2021; Naeem et al., 2022; Feng et al., 2024). Studies have shown that some natural compounds can ameliorate EMs by precisely regulating different PCD pathways (Figure 2). This review systematically integrates the latest progress in this field and describes the mechanism by which natural products regulate PCD to treat EMs.

## 2 Methods

### 2.1 Search strategy

This study systematically searched literature published between January 2015 and October 2025 in PubMed, Web of Science, and CNKI databases concerning the modulation of PCD in EMs by natural products. The search strategy was constructed using a combination of exploded medical subject headings (MeSH) and free-text terms to ensure comprehensive coverage, based on four core concepts combined with the Boolean operator “AND”: (1) EMs-related terms: “Endometriosis” OR “EMs”; (2) PCD types: “Apoptosis” OR “Autophagy” OR “Ferroptosis” OR “Pyroptosis” OR “Necroptosis” OR “Programmed Cell Death” OR “PCD”; (3) natural products: “natural products” OR “Biological Products” OR “Phytochemicals” OR “Plant Extracts” OR “Herbal Medicine” OR “Chinese Herbal” OR “Herbal extracts” OR “Plant-derived”; (4) mechanistic research: “signaling pathway” OR “mechanism”.

### 2.2 Inclusion and exclusion criteria

Study selection followed pre-established criteria. The inclusion criteria were as follows: (1) study type: *in vitro*/*in vivo* experimental studies; (2) research content: investigation of the regulatory effects on at least one form of PCD (apoptosis, autophagy, ferroptosis, pyroptosis, or necroptosis) in the context of EMs, with exploration of underlying molecular mechanisms; (3) intervention: natural products, including pure metabolites or plant extracts; (4) outcome measures: studies reporting specific signaling pathway mechanisms. The exclusion criteria comprised: (1) study type: reviews, commentaries, and conference abstracts; (2) research content: there is no research on PCD mechanisms in EMs; (3) intervention: natural products or its derivatives were not used; (4) mechanism research: no experiments to verify specific pathways; (5) accessibility: articles with unavailable full text; (6) data uniqueness: repeated articles.

### 2.3 Study screening process

**FIGURE 3:**
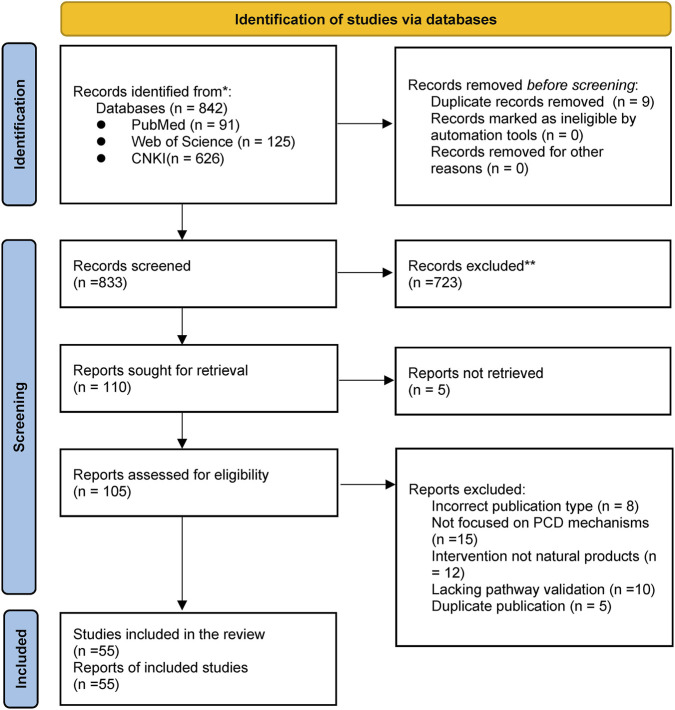
PRISMA flow diagram.

We summarize the literature screening process in the PRISMA flow diagram (Figure 3). All the articles found were imported into EndNote to remove duplicates. Two researchers independently screened the studies: first, we looked at the titles and abstracts to determine whether they met the eligibility criteria. After that, read the full text on screen. If we have different opinions during the inspection, we will discuss together or ask the third researcher until we reach an agreement. After such rigorous steps, we finally selected 55 studies.

### 2.4 Critical quality assessment framework

We used the following criteria: (1) Pan-assay interference compounds (PAINS) risk assessment: According to Bolz et al., we assessed the non-specific interference risk of each natural product due to its chemical structure (Bolz et al., 2021). High risk: the compound contains a well-defined PAINS substructure (e.g., catechol and rhodanine), which is closely associated with high false-positive rates. Medium risk: the compound exhibits potential PAINS-like reactive features. Low risk: no known PAINS substructures were identified. (2) Evidence level classification: The strength of evidence supporting the anti-EMs activity of each compound was graded according to the study design type and rigor of validation. Level 1: evidence from clinical randomized controlled trials. Level 2: pharmacodynamic evidence from *in vivo* animal models, accompanied by mechanistic exploration. Level 3: evidence verified by multiple complementary *in vitro* assays. Level 4: evidence based on a single or preliminary *in vitro* assay. Particularly, we emphasize that for compounds assessed as “high risk”, the credibility of their reported activity (including *in vivo* evidence) is significantly diminished, and the related conclusions must be interpreted with caution.

### 2.5 Data extraction and synthesis

Standardized data extraction and comprehensive analysis were performed for all included studies. The key information extracted included: the name and source of the natural product, study model, dosage, main biological effects and related signaling pathways, type of extract, experimental control settings, and toxic side effects. Subsequently, we analyzed the strength of evidence for each study and annotated its PAINS risk level and evidence level. The detailed content is summarized in Tables 1, 2 and Supplementary Tables S1, S2.

**TABLE 1:** Natural products target apoptosis for treating EMs.

Natural products	Origination	Type of study	Models	Dosage	Minimal effective dose	Biological effects	Results	References
Ginsenoside Rg3	*Panax ginseng* C.A.Mey. [Araliaceae]	*In vivo*	SD rats	5, 10 mg/kg	5 mg/kg	↓: p-Akt, p-mTOR, E_2_, P, VEGF, VEGFR-2	Induce apoptosis, inhibit angiogenesis, and reduce lesion volume	Cao et al. (2017b)
Chrysin	Propolis (a resin mixture collected by bees), or *Passiflora incarnata* L. [Passifloraceae]	*In vitro*	End1/E6E7, VK2/E6E7	0, 5, 10, 20, 50, 100 µM	20 μM	↓: p-AKT ↑: ROS	Inhibit proliferation, block the cell cycle, and induce apoptosis	Ryu et al. (2019)
Apigenin	*Apium graveolens* L. [Apiaceae]	*In vitro*	End1/E6E7, VK2/E6E7	0, 5, 10, 20 µM	5 µM	↓: AKT, ERK1/2, JNK ↑: Bax, Bak, Cyt-c, ROS	Induce apoptosis, inhibit proliferation, and block the cell cycle	Park et al. (2018)
Quercetin	Onions (*Allium cepa* L., [Amaryllidaceae]), Apples (*Malus domestica* Borkh., [Rosaceae]), Chili peppers (*Capsicum annuum* L., [Solanaceae]), etc	*In vitro*/*In vivo*	*In vitro*: End1/E6E7, VK2/E6E7; *In vivo*: C57BL/6 mice	*In vitro*: 0, 2, 5, 10, 20, 50 μM; *In vivo*: 35 mg/kg	*In vitro*: 20 μM; *In vivo*: 35 mg/kg	↓: AKT, ERK1/2, P90RSK, P70S6K ↑: ROS	Suppress inflammation, induce apoptosis, inhibit proliferation, block the cell cycle, and reduce lesion volume	Park et al. (2019a), Delenko et al. (2024)
Fraxetin	*Fraxinus chinensis* Roxb. [Oleaceae]	*In vitro*/*In vivo*	*In vitro*: End1/E6E7, VK2/E6E7; *In vivo*: C57BL/6 mice	*In vitro*: 0, 5, 10, 20, 50, 100 μM; *In vivo*: 30 mg/kg	*In vitro*: 20 μM; *In vivo*: 30 mg/kg	↓: Bcl-2, p-JNK, p-ERK1/2, p-P38, p-AKT ↑: Bax, Bak	Induce apoptosis, inhibit proliferation, inhibit cell migration and invasion, and reduce lesion volume	Ham et al. (2024)
Baicalein	*Scutellaria baicalensis* Georgi [Lamiaceae]	*In vitro*/*In vivo*	*In vitro*: Immortalized human ovarian endometriotic stromal cells; *In vivo*: C57BL/6 mice	*In vitro*: 0.5–100 μg/mL; *In vivo*: 40 mg/kg	*In vitro*: 1–2 μg/mL; *In vivo*: 40 mg/kg	↓: p-MAPK, p-JNK, p-ERK1/2, p-P70S6K, p-S6, GPX1, SOD1 ↑: Bax, BAD, BAK, caspase-1	Induce apoptosis, inhibit proliferation, block the cell cycle, and reduce lesion volume	Park et al. (2024)
Ursolic acid	*Rosmarinus officinalis* L. [Lamiaceae]	*In vitro*	Primary human endometriotic stromal cells	15, 30, 45, 60 μM	30 µM	↓: COX-2, PGE2, VEGF ↑: caspase-3, p-JNK/t-JNK, p-p38/t-p38	Induce apoptosis, inhibit proliferation, and inhibit angiogenesis	Li et al. (2020)
Silibinin	*Silybum marianum* (L.) Gaertn. [Asteraceae]	*In vivo*	Wistar rats	50 mg/kg	50 mg/kg	↓: Bcl-2, Bcl-6b ↑: ERK1/2	Induce apoptosis, inhibit angiogenesis, and reduce lesion volume	Nahari and Razi (2018)
		*In vitro*/*In vivo*	*In vitro*: End1/E6E7, VK2/E6E7; *In vivo*: C57BL/6 mice	*In vitro*: 0, 5, 10, 25 μM; *In vivo*: 100 mg/kg	*In vitro*: 25 μM; *In vivo*: 100 mg/kg	↑: JNK/t-JNK, p-p38/t-p38	Suppress inflammation, induce apoptosis, inhibit proliferation, and reduce lesion volume	Ham et al. (2019)
Naringenin	*Citrus* × *paradisi* Macfad. [Rutaceae]	*In vitro*	End1/E6E7, VK2/E6E7	0, 5, 10, 20, 50, 100 μM	100 µM	↓: p-AKT, p-ERK1/2, P90RSK, Bcl-2 ↑: p-JNK, P38, Bax, Bak, ROS	Induce apoptosis and inhibit proliferation	Park et al. (2017)
Luteolin	*Brassica oleracea* L. [Brassicaceae]	*In vitro*/*In vivo*	*In vitro*: End1/E6E7, VK2/E6E7; *In vivo*: C57BL/6 mice	*In vitro*: 0, 5, 10, 20, 50, 100 μM; *In vivo*: 40 mg/kg	*In vitro*: 20 μM; *In vivo*: 40 mg/kg	↓: ERK1/2, JNK, PI3K/AKT ↑: P38	Induce apoptosis, inhibit proliferation, and reduce lesion volume	Park et al. (2019c)
Delphinidin	*Solanum melongena* L. [Solanaceae]	*In vitro*	End1/E6E7, VK2/E6E7	0, 20, 50, 100 μM	100 µM	↓: ERK1/2, AKT, P70S6K ↑: P38 MAPK	Induce apoptosis, inhibit proliferation, and block the cell cycle	Park et al. (2019b)
Myricetin	*Morella rubra* Lour. [Myricaceae]	*In vitro*/*In vivo*	*In vitro*: End1/E6E7, VK2/E6E7; *In vivo*: C57BL/6 mice	*In vitro*: 0, 5, 10, 20, 50, 100 μM; *In vivo*: 30 mg/kg	*In vitro*: 20 μM; *In vivo*: 30 mg/kg	↓: AKT, ERK1/2 ↑: ROS, P38	Induce apoptosis, inhibit proliferation, block the cell cycle, and reduce lesion volume	Park et al. (2020)
Baicalein	*Scutellaria baicalensis* Georgi [Lamiaceae]	*In vitro*	Human endometrial stromal cells	0, 5, 10, 20, 40, 80, 160 µM	5 µM	↓: Bcl-2, NF-κB	Induce apoptosis, inhibit proliferation, and block the cell cycle	Jin et al. (2017)
Curcumin	*Curcuma longa* L. [Zingiberaceae]	*In vivo*	Balb/C mices	12, 24, 48 mg/kg	48 mg/kg	↓: NF-κB ↑: Bax/Bcl-2, caspase-9	Induce apoptosis, inhibit proliferation, and reduce lesion volume	Cao et al. (2017a)
Ginsenoside Rg3	*Panax ginseng* C.A.Mey. [Araliaceae]	*In vitro*	Human ectopic endometrial stromal cells	0, 25, 50, 100, 150 μg/mL	100 μg/mL	↓: NF-kB, p65, VEGF ↑: caspase-3	Induce apoptosis, inhibit proliferation, and inhibit angiogenesis	Huang et al. (2020)
Dehydrocostus lactone	*Aucklandia lappa* Decne. [Asteraceae]	*In vitro*	Human endometriotic 12Z cells	1, 2, 5, 10, 20 μM	5 µM	↓: NF-κB, AKT, Bcl, IL-10, VEGF, MMP-2, MMP-9 ↑: caspase-3, caspase-8, caspase-9	Suppress inflammation, induce apoptosis, inhibit proliferation, and relieve pain	Woo et al. (2019)
Flavokawain A	*Piper methysticum* G.Forst. [Piperaceae]	*In vivo*	Sprague-Dawley rats	10, 50 mg/kg	50 mg/kg	↓: NF-κB, PI3K, p-PI3K, AKT, p-AKT, Bcl-2, IL-1β, IL-6, IL-8, NO, COX-2, TNF-α, PGE2, VEGF ↑: Bax, caspase-3	Suppress inflammation, induce apoptosis, inhibit angiogenesis, and reduce lesion volume	Wei et al. (2024)
Curcumin	*Curcuma zedoaria* (Christm.) Roscoe [Zingiberaceae]	*In vitro*/*In vivo*	*In vitro*: Ectopic endometriotic stromal cells; *In vivo*: Sprague-Dawley rats	*In vitro*: 2.5–40 μg/mL; *In vivo*: 20 mg/kg	*In vitro*: 5 μg/mL; *In vivo*: 20 mg/kg	↓: JAK2, STAT3, Bcl-2, TNF-α, IL-6, IL-1β ↑: Bax, caspase-3	Suppress inflammation, induce apoptosis, inhibit proliferation, inhibit cell migration and invasion, and reduce lesion volume	Wang et al. (2022)
β-Sitosterol	Ubiquitous in the plant kingdom, e.g., Soybean (*Glycine max* (L.) Merr., [Fabaceae]), corn germ oil, wheat germetc.	*In vitro*/*In vivo*	*In vitro*: Ectopic endometrial stromal cell line (hEM15A); *In vivo*: C57BL/6 mice	*In vitro*: 30, 60, 90 μM; *In vivo*: 35, 350 μg/kg	*In vitro*: 30 μM; *In vivo*: 35 μg/kg	↓: TGF-β1, Smad2, Smad3 ↑: smad7	Induce apoptosis, inhibit proliferation, inhibit cell migration and invasion, and reduce lesion volume	Wen et al. (2023)
Tanshinone IIA	*Salvia miltiorrhiza* Bunge [Lamiaceae]	*In vivo*	SD rats	30 mg/kg	30 mg/kg	↓: TGF-β1, p-SMAD2, p-SMAD3, Bcl-2, VEGF, MMP9 ↑: SMAD7, caspase9, Bax	Induce apoptosis and reduce lesion volume	Ma et al. (2021)
Picroside II	*Neopicrorhiza scrophulariiflora* (Pennell) D.Y.Hong [Plantaginaceae]	*In vivo*	SD rats	5, 10, 30 mg/kg	10 mg/kg	↓: Bcl-2, VEGF, TGF-β	Induce apoptosis, inhibit angiogenesis, and reduce lesion volume	Li et al. (2020)
Rutin	*Fagopyrum esculentum* Moench [Polygonaceae]	*In vitro*/*In vivo*	*In vitro*: Ectopic endometrial stromal cell line (CRL-7566); *In vivo*: Wistar rats	*In vitro*: 0, 30, 50, 70, 90 μM; *In vivo*: 3, 6 mg/kg	*In vitro*: 70 μM; *In vivo*: 3 mg/kg	↓: ROS, NOX4, HIF-1α, Bcl2, Ki-67, MMP2, MMP9, p-mTOR, MDA ↑: Bax, caspase-9, GPX, SOD	Induce apoptosis, inhibit proliferation, inhibit cell migration and invasion, and reduce lesion volume	Talebi et al. (2021), Wang et al. (2023)
Pro-EGCG	A derivative of Epigallocatechin gallate (typically synthesized from EGCG found in tea (*Camellia sinensis* (L.) Kuntze, [Theaceae])	*In vitro*/*In vivo*	*In vitro*: Human endometriotic cell line, HS293; *In vivo*: C57BL/6 mice	*In vitro*: 0–300 μM; *In vivo*: 25, 50 mg/kg	*In vitro*: 100 μM; *In vivo*: 25 mg/kg	↓: HIF-1a,VEGF	Induce apoptosis, inhibit proliferation, inhibit angiogenesis, and reduce lesion volume	Hung et al. (2024)
Resveratrol	*Vitis vinifera* L. [Vitaceae]	*In vitro*/*In vivo*	*In vitro*: Human ectopic endometrial stromal cells; *In vivo*: Sprague-Dawley rats	*In vitro*: 40, 100 μM; *In vivo*: 15, 45 mg/kg	*In vitro*: 40 μM; *In vivo*: 15 mg/kg	↓: Bcl-2, MMP-2, VEGF ↑: Bax, caspase-3	Induce apoptosis, inhibit proliferation, inhibit cell migration and invasion, inhibit angiogenesis, and reduce lesion volume	Chen et al. (2021b)
Isoliquiritigenin	*Glycyrrhiza uralensis* Fisch. ex DC. [Fabaceae]	*In vitro*/*In vivo*	*In vitro*: End1/E6E7; *In vivo*: Balb/C mice	*In vitro*: 10, 25, 50, 75, 100 μM; *In vivo*: 1, 5 mg/kg	*In vitro*: 25 μM; *In vivo*: 1 mg/kg	↓: Bcl-2 ↑: Bax, caspase-3	Suppress inflammation, induce apoptosis, inhibit proliferation, inhibit cell migration and invasion, and reduce lesion volume	Hsu et al. (2020)
Shikonin	*Lithospermum erythrorhizon* Sieb. et Zucc. [Boraginaceae]	*In vivo*	SD rats	300, 500, 800 mg/kg	300 mg/kg	↓: Bcl-2 ↑: Bax, Bax/Bcl-2	Induce apoptosis and reduce lesion volume	Zhang and Tang (2018)
Ligustrazine	*Ligusticum striatum* DC. [Apiaceae]	*In vitro*	Ectopic endometriotic stromal cells	12, 24, 48 μM	12 µM	↓: Bcl-2, E-cadherin ↑: Bax, caspase-3, N-cadherin, MMP-9	Induce apoptosis, inhibit proliferation, and inhibit cell migration and invasion	Tang et al. (2023)
Betulinic acid	*Betula pendula* Roth [Betulaceae]	*In vitro*	Human endometrial epithelial cells; Human endometriotic 12Z cells	0–40 μM	20 μM	↓: Bcl-2, SOD2, NRF1, COX2, MMP1, IL6, TNF-α ↑: caspase-3, ROS	Suppress inflammation, induce apoptosis, inhibit proliferation, inhibit cell migration, and inhibit invasion	Xiang et al. (2020)
Naringenin	*Citrus × paradisi* Macfad. [Rutaceae]	*In vitro*/*In vivo*	*In vitro*: Human endometrial stromal cells; *In vivo*: SD rats	*In vitro*: 0–10 μM; *In vivo*: 50 mg/kg	*In vitro*: 0.5 µM; *In vivo*: 50 mg/kg	↓: Bcl-2, MMP-2, MMP-9, VEGF, TNF-α ↑: caspase-3, Cyt-c, ROS	Suppress inflammation, induce apoptosis, inhibit proliferation, inhibit cell migration and invasion, and reduce lesion volume	Kapoor et al. (2019)

Detailed experimental parameters, controls, and PAINS, risk assessments for each compound are provided in Supplementary Table S1.

**TABLE 2:** Natural products target autophagy, ferroptosis, and pyroptosis for treating EMs.

PCD type	Natural products	Origination	Type of study	Models	Dosage	Minimal effective dose	Biological effects	Results	References
Autophagy	Polyphyllin I	*Paris polyphylla* Sm. [Melanthiaceae]	*In vitro*	Ectopic endometriotic stromal cells	0.25, 0.5, 0.75, 1.0, 1.5, 2.0 µM	0.25 µM	↓: p62, Bcl-2, p-AKT, p-mTOR ↑: Beclin-1, LC3-II, Bax	Induce apoptosis, inhibit proliferation, and induce autophagy	Li et al. (2025)
Autophagy	Carvacrol	*Origanum vulgare* L. [Lamiaceae]	*In vitro*/*In vivo*	*In vitro*: Immortalized human ovarian endometriotic stromal cells; *In vivo*: C57BL/6 mice	*In vitro*: 0, 50, 100, 200 μM; *In vivo*: 100 mg/kg	*In vitro*: 20 μM; *In vivo*: 100 mg/kg	↓: p-AKT ↑: Beclin-1	Suppress inflammation, inhibit proliferation, induce autophagy, and reduce lesion volume	Jang et al. (2025)
Autophagy	Saikosaponin A	*Bupleurum chinense* DC. [Apiaceae]	*In vitro*	Human ectopic endometrial stromal cells	7.5, 10, 12.5, 15, 17.5 µM	10 µM	↓: p62, p-AKT/AKT,p-mTOR/mTOR ↑: Beclin-1, LC3-II	Induce apoptosis, inhibit proliferation, inhibit cell migration and invasion, and induce autophagy	Zhou (2024)
Autophagy	Timosaponin AIII	*Anemarrhena asphodeloides* Bunge [Asparagaceae]	*In vitro*	Human ectopic endometrial stromal cells	1, 2.5, 5, 7.5, 10, 12.5 µM	2.5 μM	↓: Bcl-2, p62, p-AKT/AKT ↑: Bax, LC3	Induce apoptosis, inhibit proliferation, inhibit cell migration and invasion, and induce autophagy	Wan (2024)
Autophagy	Alpinumisoflavone	*Erythrina lysistemon* Hutch. [Fabaceae]	*In vitro*	End1/E6E7, VK2/E6E7	0, 5, 10, 20, 50 µM	20 µM	↓: P70S6K, S6 ↑: Beclin1, ATG5	Induce apoptosis, inhibit proliferation, inhibit cell migration and invasion, and induce autophagy	Song et al. (2023)
Autophagy	Berberine	*Coptis chinensis* Franch. [Ranunculaceae] and *Phellodendron amurense* Rupr. [Rutaceae]	*In vitro*	Bovine endometrial epithelial cells	5, 10, 20 µM	10 µM	↓: p62 ↑: Bax/Bcl2, Beclin-1, LC3II, Nrf2	Induce apoptosis, inhibit proliferation, and induce autophagy	Fu et al. (2023)
Autophagy	Quercetin	*Allium cepa* L. [Amaryllidaceae]	*In vivo*	Sprague-Dawley rats	15 mg/kg	15 mg/kg	↓: mTOR, TNF-α ↑: Nrf2, Beclin-1, ATG5	Reduce lesion volume and induce autophagy	Jamali et al. (2021)
Autophagy	Protopanaxadiol	*Panax ginseng* C.A.Mey. [Araliaceae]	*In vitro*/*In vivo*	*In vitro*: Ectopic endometriotic stromal cells; *In vivo*: Balb/C mice	*In vitro*: 0–160 μM; *In vivo*: 15, 30, 45 mg/kg	*In vitro*: 30–40 µM. *In vivo*: 15 mg/kg	↓: p62, Bcl-2/Bcl-xL, Ki-67, ERα, IL-10 ↑: Beclin-1, LC3-II, Bax/Bak, PRα	Suppress inflammation, induce apoptosis, inhibit proliferation, reduce lesion volume, and induce autophagy	Zhang et al. (2018)
Autophagy	Gamma oryzanol	*Oryza sativa* L. [Poaceae]	*In vivo*	Wistar rats	3, 6 mg/kg	3 mg/kg	↑: Beclin-1, LC3II	Induce apoptosis, induce autophagy, and reduce lesion volume	Eisalou and Farahpour (2022)
Autophagy	Soy isoflavones	*Glycine max* (L.) Merr. [Fabaceae]	*In vivo*	Sprague-Dawley rats	50 mg/kg	50 mg/kg	↓: Bcl-2 ↑: Beclin-1, LC3, Bax, caspase-3	Induce apoptosis, induce autophagy, and reduce lesion volume	Sabetian et al. (2024)
Autophagy	SCM-198	*Leonurus japonicus* Houtt. [Lamiaceae]	*In vitro*/*In vivo*	*In vitro*: Ectopic endometriotic stromal cells; *In vivo*: C57BL/6 mice	*In vitro*: 0, 50, 100, 200 μM; *In vivo*: 7.5, 15 mg/kg	*In vitro*: 100 μM; *In vivo*: 7.5 mg/kg	↓: Bcl-2 ↑: Beclin-1, LC3, Bax	Induce apoptosis, induce autophagy, and reduce lesion volume	Lin et al. (2022)
Autophagy	Paeonol	*Paeonia suffruticosa* Andrews [Paeoniaceae]	*In vitro*/*In vivo*	*In vitro*: Ectopic endometriotic stromal cells; *In vivo*: SD rats	*In vitro*: 0, 10, 30, 50, 100 μM; *In vivo*: 20, 50 mg/kg	*In vitro*: 10 μM; *In vivo*: 20 mg/kg	↓: HIF1α, Beclin-1, LC3-II, IL-1β, IL-6, TNF-α	Suppress inflammation, inhibit apoptosis, inhibit autophagy, and reduce lesion volume	Pang et al. (2021)
Ferroptosis	β-elemene	*Curcuma wenyujin* Y.H.Chen and C.Ling [Zingiberaceae]	*In vitro*/*In vivo*	*In vitro*: Human endometriotic 12Z cells; *In vivo*: C57BL/6 mice	*In vitro*: 0–100 μg/mL; *In vivo*: 25, 50, 75 mg/kg	*In vitro*: 15 μg/mL; *In vivo*: 25 mg/kg	↓: p-STAT3, p-MAPK14, GPX4, GSH ↑: ROS	Inhibit proliferation, inhibit cell migration and invasion, reduce lesion volume, and induce ferroptosis	Fu et al. (2025)
Ferroptosis	Resveratrol	*Vitis vinifera* L. [Vitaceae]	*In vitro*/*In vivo*	*In vitro*: Ectopic endometriotic stromal cells; *In vivo*: Balb/C mice	*In vitro*: 0, 25, 50, 100, 200 μM; *In vivo*: 25 mg/kg	*In vitro*: 25 μM; *In vivo*: 25 mg/kg	↓: GPX4, SLC7A11, GSH ↑: ROS	Inhibit proliferation, reduce lesion volume, and induce ferroptosis	Zou et al. (2024)
Ferroptosis	Ginsenoside Rf	*Panax ginseng* C.A.Mey. [Araliaceae]	*In vitro*/*In vivo*	*In vitro*: Endometriotic stromal cells; *In vivo*: Wistar rats	*In vitro*: 20 μM; *In vivo*: 1, 2, 3 mg/kg	*In vitro*: 20 μM; *In vivo*: 1 mg/kg	↓: GPX4, SLC7A11 ↑: Beclin-1, LC3-II/I	Inhibit proliferation, reduce lesion volume, induce autophagy, and induce ferroptosis	Zhang L. et al. (2024)
Ferroptosis	Baicalein	*Scutellaria baicalensis* Georgi [Lamiaceae]	*In vitro*	The monocyte cell line THP1	20 µM	20 µM	↑: GPX4	Inhibit ferroptosis	Yi et al. (2022)
Ferroptosis	Pachymic acid	*Wolfiporia cocos* (Schwein.) Ryvarden and Gilb. [Polyporaceae]	*In vitro*/*In vivo*	*In vitro*: Ectopic endometriotic stromal cells; *In vivo*: SD rats	*In vitro*: 40, 80 mg/L; *In vivo*: 3.5, 7 mg/kg	*In vitro*: 40 mg/L; *In vivo*: 3.5 mg/kg	↓: TNF-α, IL-6 ↑: Nrf2, p-AMPK/AMPK, p-GSK-3β/GSK-3β, GSH	Suppress inflammation, induce apoptosis, inhibit proliferation, reduce lesion volume, and inhibit ferroptosis	Li et al. (2024)
Ferroptosis	Wogonin	*Scutellaria baicalensis* Georgi [Lamiaceae]	*In vivo*	SD rats	7, 14 mg/kg	7 mg/kg	↓: IL-1β, IL-6 ↑: Nrf2, GPX, SIRT1, FTL, SLC7A11	Suppress inflammation, reduce lesion volume, and inhibit ferroptosis	Hu et al. (2023)
Pyroptosis	Fisetin	*Rhus typhina* L. [Anacardiaceae]	*In vivo*	Sprague-Dawley rats	40 mg/kg	40 mg/kg	↓: NLRP-3, caspase-1, Bcl-2, NF-κB, IL-1β, TNF-α ↑: Bax, caspase-3	Suppress inflammation, inhibit pyroptosis, and reduce lesion volume	Arangia et al. (2023)
Pyroptosis	Tetramethylpyrazine	*Ligusticum striatum* DC. [Apiaceae]	*In vivo*	Sprague-Dawley rats	8, 19, 38 mg/kg	8 mg/kg	↓: NLRP3, caspase-1, GSDMD-N, TNF-α, IL-6, IL-2, IL-10 ↑: Nrf2	Suppress inflammation, inhibit pyroptosis, and reduce lesion volume	Xu et al. (2025)
Pyroptosis	CHS-Iva	*Panax japonicus* (T.Nees) C.A.Mey. [Araliaceae]	*In vitro*/*In vivo*	*In vitro*: Ectopic endometriotic stromal cells; *In vivo*: C57BL/6 mice	*In vitro*: 0, 5, 10, 50, 100, 200 μM; *In vivo*: 50 mg/kg	*In vitro*: 10 μM; *In vivo*: 50 mg/kg	↓: NLRP3, caspase-1, GSDMD, IL-1β, IL-6, TNF-α	Suppress inflammation, inhibit pyroptosis, and reduce lesion volume	Huang et al. (2024b)
Pyroptosis	Curcumin	*Curcuma longa* L. [Zingiberaceae]	*In vitro*/*In vivo*	*In vitro*: Ectopic endometriotic stromal cells; *In vivo*: C57BL/6 mice	*In vitro*: 5–80 μM; *In vivo*: 200 mg/kg	*In vitro*: 10 μM; *In vivo*: 200 mg/kg	↓: NLRP3, caspase-1, ASC, GSDMD, IL-1β, IL-18	Suppress inflammation, inhibit pyroptosis, and reduce lesion volume	Ding et al. (2024)
Pyroptosis	Paeonol	*Paeonia × suffruticosa* Andrews [Paeoniaceae]	*In vitro*/*In vivo*	*In vitro*: Mice endometriotic epithelial cells; *In vivo*: Bally/c mouse	*In vitro*: 50, 100 μM; *In vivo*: 12.5, 25, 50 mg/kg	*In vitro*: 50 μM; *In vivo*: 12.5 mg/kg	↓: NLRP3, ASC, caspase-1, GSDMD-N, IL-6, TNF-α, IL-1β, IL-18 ↑: Nrf2, HO-1, NQO-1	Suppress inflammation, inhibit proliferation, inhibit cell migration and invasion, inhibit pyroptosis, and reduce lesion volume	Du et al. (2024)

Detailed experimental parameters, controls, and PAINS risk assessments for each compound are provided in Supplementary Table S2.

## 3 Mechanism of action of PCD in EMs

### 3.1 Apoptosis

Apoptosis is a form of PCD essential for maintaining homeostasis, and is regulated by the extrinsic (death receptor) and intrinsic (mitochondrial) pathways (Xu et al., 2019). This regulatory network dysregulates in EMs. Studies have shown that compared with normal endometrial tissues, ectopic endometrial tissues in EMs are resistant to apoptosis. This feature allows ectopic endometrial cells to evade clearance and proliferate within the ectopic environment (Depalo et al., 2009).

Research indicates that resistance to apoptosis in EMs manifests as a synergistic dysregulation of dual pathways. In the mitochondrial pathway, Bcl-2 overexpression and reduced Bax in ectopic endometrial tissue disrupt the Bcl-2/Bax balance. This inhibits the release of cytochrome c (Cyt-C), thus inhibiting caspase-9 (Harada et al., 2004). In the death receptor pathway, ectopic cells evade immune surveillance by upregulating FasL and TNF-α, thereby inducing T-cell apoptosis through caspase-8 (Garcia-Velasco and Arici, 2003). This dual pathway dysfunction ultimately impairs caspase-3 and inhibits apoptosis. Notably, the microenvironment surrounding ectopic lesions is rich in inflammatory mediators and oxidative stress, further reinforcing resistance to apoptosis (Depalo et al., 2009).

**FIGURE 4:**
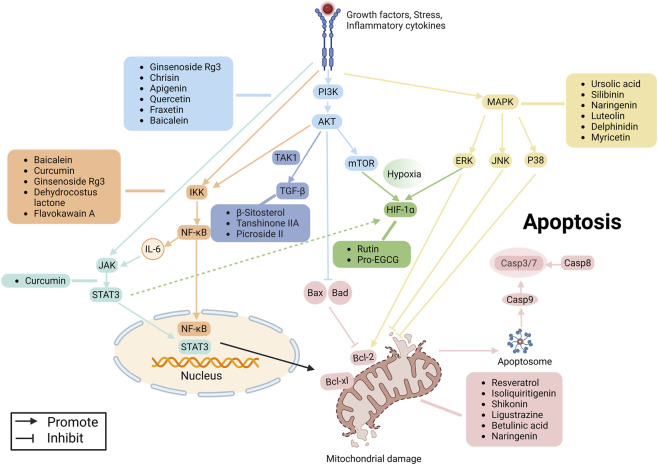
Natural products target PI3K/Akt, MAPK, NF-κB, JAK/STAT, and other signaling pathways to modulate apoptosis for treating EMs. Created with Biorender.com.

Multiple signaling pathways form a regulatory network that drives apoptosis resistance in EMs. Inflammatory and growth factors activate phosphoinositide 3-kinase/protein kinase B (PI3K/Akt) and the mitogen-activated protein kinase (MAPK). MAPK pathway resists apoptosis by activating ERK and inhibiting JNK/p38, and cooperates with PI3K/Akt to regulate apoptosis (Bora and Yaba, 2021). Activated Akt promotes ectopic endometrial cell growth by phosphorylating Bad and upregulating Bcl-2. It also activates IκB kinase (IKK), leading to nuclear factor-κB (NF-κB) translocation (Hung et al., 2021). Activated NF-κB upregulates pro-inflammatory factors IL-6 and anti-apoptotic proteins Bcl-2 and c-FLIP, thus inhibiting apoptosis (González-Ramos et al., 2010). IL-6 further activates Janus kinase/signal transducer and activator of transcription (JAK/STAT) pathway. Phosphorylated STAT3 is transferred to the nucleus and cooperates with NF-κB to promote Bcl-2 expression. The stability of hypoxia-inducible factor 1-α (HIF-1α) is reinforced by PI3K/Akt, MAPK, and STAT3, further enhancing adaptivity to hypoxic microenvironments. Furthermore, PI3K/Akt and transforming growth factor beta (TGF-β) interact through TAK1, which induces epithelial-mesenchymal transition (EMT), promoting ectopic endometrial cell invasion, fibrosis, and survival. Ultimately, this integrated network increases Bcl-2 and Bcl-xL levels, inhibiting Cyt-C release and caspase activation, thereby inducing apoptosis resistance in ectopic endometrial cells (Edalatian Kharrazi et al., 2024). Research indicates that natural products have unique advantages in regulating EMs apoptosis. For example, natural products such as apigenin (Park et al., 2018) and baicalein (Park et al., 2024) can simultaneously inhibit PI3K/Akt and MAPK, reduce Bcl-2, and reverse EMs resistance to apoptosis. So, targeting signaling molecules that modulate apoptosis may be a promising way for treating EMs (Figure 4).

### 3.2 Autophagy

Autophagy, as an evolutionarily highly conserved intracellular degradation pathway, maintains cellular homeostasis by clearing damaged organelles and proteins through the autophagosome-lysosome pathway. This process has four stages: initiation, elongation, maturation, and degradation (Kocaturk et al., 2019). Autophagy shows a dynamic dual regulatory characteristic in EMs. In the early stage of EMs, reduced autophagic activity promotes ectopic endometrial cell survival; In the late stage of EMs, autophagy activation maintains lesion survival by enhancing the adaptability of ectopic cells. Therefore, autophagy shows a “double-edged sword” characteristic at different stages of EMs.

In the early stages of EMs, autophagy is inhibited, manifested by decreased Beclin-1 and LC3. This impedes the clearance of endometrial debris returning to the abdominal cavity. High estrogen and progesterone resistance in EMs inhibits autophagy (Shen et al., 2021). Chronic inflammatory environment inhibits autophagy by releasing TNF-α and IL-6, further reducing Beclin-1 and LC3B expression (Huang et al., 2023). In the late stage, autophagic activity increases, manifested by elevated LC3-II and LC3-II/LC3-I. This is an adaptive response of ectopic cells to hypoxia and oxidative stress. Activated autophagy helps ectopic endometrial cells survive in hypoxia by recovering intracellular nutrients and energy (Yang et al., 2017).

**FIGURE 5:**
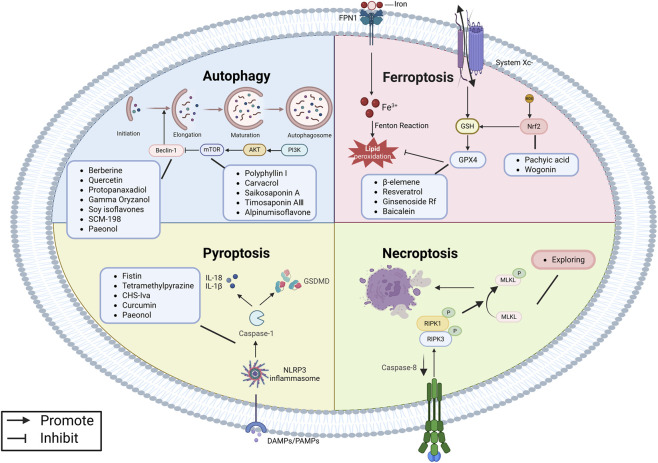
Natural products target signalling pathways such as PI3K/Akt/mTOR, Beclin-1, GPX4, Nrf2, NLRP3, RIPK1, and RIPK3 to modulate diverse PCD such as autophagy, ferroptosis, pyroptosis, and necroptosis for treating EMs. Created with Biorender.com.

Multiple signalling pathways collectively regulate autophagy. Among these, the mechanical/mammalian target of rapamycin (mTOR) is pivotal in the PI3K/Akt pathway. Research indicates that activated PI3K/Akt in EMs can stimulate mTOR, then suppress ATG to inhibit autophagy. The mTOR inhibitor rapamycin may block this inhibition and reduce angiogenesis and ectopic lesion size by activating autophagy (Siracusa et al., 2021). Concurrently, Beclin-1, which interacts with various ATGs, is regulated by estrogen and inflammatory factors, and is a key node in the autophagy network (Kong and Yao, 2022). Given the dual regulatory role of autophagy in EMs, targeted intervention strategies require precise timing. In the early stage, activating autophagy can promote ectopic cell death; In the late stage, inhibiting autophagy can prevent lesion survival. Natural products show unique advantages in regulating EMs autophagy. Natural products such as carvacrol (Jang et al., 2025) and quercetin (Jamali et al., 2021) can regulate autophagy by synergistically modulating the PI3K/Akt/mTOR signalling pathway and Beclin-1, laying a theoretical foundation for treating EMs (Figure 5).

### 3.3 Ferroptosis

Ferroptosis is an iron-dependent form of PCD characterised by lipid peroxidation, which plays an important role in EMs pathogenesis (Cheng et al., 2025). Iron overload in peritoneal fluid and ectopic lesions of EMs promotes ferroptosis (Ng et al., 2020). Ferroptosis modulates EMs through two interrelated pathways. The extrinsic one promotes ferroptosis by blocking the glutamate-cystine antiporter system Xc to restrict cystine uptake or inhibiting ferroportin 1 (FPN1) to increase intracellular iron levels. The intrinsic pathway relies on glutathione peroxidase 4 (GPX4) to prevent lipid peroxidation, thereby inhibiting ferroptosis (Chen X. et al., 2021).

Ferroptosis shows a unique dual regulatory role in EMs pathogenesis (Cheng et al., 2025). Iron overload of EMs triggers lipid peroxidation through the Fenton reaction, thereby eliminating ectopic endometrial cells (Wyatt et al., 2023). Surviving ectopic endometrial cells resist ferroptosis by activating antioxidant mechanisms (Li et al., 2022). Given this characteristic, therapy needs vary with different disease stages (Lan et al., 2025). In the early disease stage, ferroptosis inducer Erastin eliminates ectopic endometrial stromal cells (EESCs) by promoting intracellular iron accumulation and lipid peroxidation (Li et al., 2021). Conversely, in advanced stages of EMs, ferroptosis inhibitors ferrostatin-1 can reduce reactive oxygen species (ROS) and malondialdehyde (MDA) levels, alleviating oxidative stress and decreasing fibrosis in ectopic tissues (Li et al., 2021). This stage-specific therapeutic approach provides new ideas for treating EMs.

Multiple molecular pathways collectively regulate ferroptosis. As a key negative regulator of ferroptosis, GPX4 plays a central role in sustaining ectopic cell survival by reducing lipid peroxides. Research indicates that inhibiting GPX4 reduces ectopic cell resistance to ferroptosis, providing a crucial therapeutic target (Huang et al., 2025). The Nrf2 signalling pathway is the core regulator of the antioxidant defence system, which enhances cellular antioxidant capacity by upregulating GPX4 and SLC7A11, thereby inhibiting ferroptosis. This offers a new strategy for treating EMs (Xiang et al., 2024). Natural products such as pachyic acid (Li et al., 2024) and wogonin (Hu et al., 2023) offer a new therapeutic approach for regulating ferroptosis by modulating GPX4 or the Nrf2 signalling pathway (Figure 5).

### 3.4 Pyroptosis

Pyroptosis is an inflammatory form of cell death mediated by gasdermin proteins. It is characterized by cell swelling, pore formation in the cell membrane, and the membrane will rupture, releasing a large amount of intracellular contents, which is closely associated with the chronic inflammatory microenvironment of EMs (Vasudevan et al., 2023). The classic NLRP3/caspase-1/GSDMD pathway is abnormally activated in EMs. Many studies have found that key pyroptosis proteins NLRP3, caspase-1, GSDMD, and inflammatory factors IL-1β and IL-18 are elevated in ectopic tissues and peritoneal fluid of EMs (An et al., 2024).

Pyroptosis promotes disease progression in several ways in EMs. Inflammatory mediators released by pyroptotic cells activate the NF-κB pathway, establishing a positive inflammatory feedback. This persistent inflammatory environment promotes ectopic endometrial cell growth, fibrosis, and angiogenesis (Xu et al., 2023; Zhang et al., 2023). Notably, interactions between macrophages and EESCs activate the NLRP3 inflammasome. This process increases IL-1β and turns macrophages into pro-inflammatory M1 type, which aggravates immune dysregulation by disrupting T lymphocyte balance (Zhou et al., 2022). NLRP3 inhibitors prevent these pathological effects (Guo et al., 2023). Mitochondrial dysfunction in ectopic endometrial cells promotes ROS production. These activate the NLRP3 inflammasome and promote GSDMD activation, thereby promoting EMs progression (Irandoost et al., 2023).

Given the role of pyroptosis in promoting inflammation in EMs, targeting NLRP3 and GSDMD has become a promising therapeutic strategy (Bullon and Navarro, 2017). Abnormal activation of NLRP3 and GSDMD promotes the release of inflammatory mediators, leading to destruction of the immune microenvironment and metabolic dysfunction (Miao et al., 2023). Research indicates that natural products have significant potential in regulating pyroptosis. For example, curcumin (Ding et al., 2024) and paeonol (Du et al., 2024) inhibit NLRP3 and GSDMD. Therefore, using natural products to target pyroptosis provides a promising method for EMs treatment (Figure 5).

### 3.5 Necroptosis

Necroptosis is a regulated inflammatory cell death. It plays an important role in various diseases, including cancer, neurodegenerative conditions, ischemic diseases, and inflammatory disorders (Hu et al., 2022; Yan et al., 2022; Balusu and De Strooper, 2024; Mei et al., 2024). It is regarded as a cellular defense mechanism and plays a complex role in EMs. When caspase-8 is inhibited, RIPK1-RIPK3-MLKL signalling activates. Active RIPK1 interacts with RIPK3 and phosphorylates it. Activated RIPK3 in turn activates MLKL. This series of reactions causes cell swelling, cell membrane rupture, and the release of various damage-related molecular patterns (DAMPs), similar to the inflammatory state observed in EMs (Wang X. et al., 2023). Research indicates that inflammatory mediators and oxidative stress in EMs may activate necroptosis, while DAMPs further promote inflammatory responses, creating a vicious cycle. This self-reinforcing inflammatory environment promotes ectopic endometrial cell growth and influences immune cell function. Notably, necroptosis may be related to chronic pelvic pain and fibrosis of EMs. However, the exact relationship between necroptosis and EMs is unclear. Its differential expression in ectopic versus eutopic endometrium, correlation with disease staging, and interactions with other forms of cell death warrant in-depth investigation. RIPK3 is an important molecular switch that determines whether necroptosis occurs, while MLKL directly executes necroptosis. Therefore, regulating necroptosis by targeting RIPK1, RIPK3, or MLKL provides new therapeutic approaches for EMs (Figure 5).

## 4 Natural products prevent EMs by regulating PCD

### 4.1 Natural products regulating apoptosis for EMs

Recent studies have confirmed that natural compounds can effectively prevent the development of EMs by regulating key signaling pathways, including PI3K/Akt, MAPK, NF-κB, JAK/STAT, TGF-β, HIF-1α, and Bcl-2. These findings provide reliable theoretical support for treating EMs with natural products.

#### 4.1.1 Targeting the PI3K/Akt signaling pathway

The PI3K/Akt signaling pathway is a key mechanism controlling apoptosis through multi-target phosphorylation. As an anti-apoptotic regulator, activated Akt prevents apoptosis in several ways. On one hand, active Akt enhances anti-apoptotic function by activating the regulatory protein Bcl-2 in the intrinsic apoptotic pathway. On the other hand, Akt can directly inhibit caspase-9 activation, effectively interrupting the apoptosis process (Yu et al., 2025). Using natural products to inhibit the PI3K/Akt pathway and induce apoptosis may offer a novel therapeutic strategy for EMs.

Ginsenoside Rg3 is a triterpene derived from *Panax ginseng* C.A. Mey. [Araliaceae]. At 5 and 10 mg/kg, ginsenoside Rg3 minimized endometrial lesions. It reduced p-Akt and p-mTOR levels, thereby relieving the inhibition of downstream pro-apoptotic signals (Cao Y. et al., 2017). However, this study lacks toxicity assessment and key pharmacokinetic data. Future research should conduct safety assessments and pharmacokinetic studies to provide sufficient evidence for clinical translation.

Chrysin is a naturally occurring flavonoid found in *Passiflora incarnata* L. [Passifloraceae]. At 20 μM, chrysin inhibited End1/E6E7 and VK2/E6E7 proliferation and induced apoptosis. It also regulated endoplasmic reticulum (ER) stress, increased calcium ion concentration, and promoted ROS production. It probably did this through the PI3K pathway suppression (Ryu et al., 2019). However, this study uses a simplified model of immortal cell lines that lacks the key pathological environment of EMs. This study did not conduct animal experiments to test the bioavailability, efficacy, and safety of chrysin.

Apigenin is a trihydroxyflavone present in many fruits, including celery, apples, and grapes. At concentrations of 0, 5, 10, and 20 μM in VK2/E6E7 and End1/E6E7 cells, apigenin significantly increased the levels of Bax, Bak, Cyt-c, ROS, and calcium ions, effectively inducing apoptosis. This effect was associated with downregulation of Akt, ERK1/2, and JNK (Park et al., 2018). However, this study uses a simplified model of immortal cell lines that lacks the key pathological environment of EMs. No animal studies have verified apigenin’s bioavailability and toxicity. Future research should validate its efficacy and safety in animal models while enhancing its bioavailability.

Quercetin is a flavonol. Animal and cell studies have shown that quercetin could upregulate Akt, ERK1/2, p38MAPK, P90RSK, and P70S6K, promote ROS production, and alter mitochondrial membrane potential. In this way, it inhibited the growth of VK2/E6E7 and End1/E6E7 cells, induced apoptosis, caused cell cycle arrest, and reduced proliferation and inflammation in EMs mice. These beneficial effects may stem from inhibition of the PI3K and MAPK pathways (Park et al., 2019a). Laboratory studies have also shown that quercetin can inhibit EESC proliferation via the Akt–ERK–p53 signaling pathway, promote cell differentiation, and accelerate senescent cell death (Delenko et al., 2024). However, these results have not yet been validated in humans. Quercetin is a high-risk PAINS compound; these findings require cautious interpretation. The primary constraints to clinical translation lie in the lack of human pharmacokinetic, safety, and efficacy data, coupled with quercetin’s low oral bioavailability.

Fraxetin is a coumarin derivative derived from *Fraxinus chinensis* Roxb. [Oleaceae]. In an EMs mouse model, 30 mg/kg fraxetin for 28 days reduced adhesion and upregulated Bax and Bad levels. 50 and 100 μM fraxetin inhibited End1/E6E7 and VK2/E6E7 cell growth, induced ROS production, triggered ER stress, downregulated Bcl-2, p-JNK, and p-Akt, and upregulated Bak and Bax. In other words, fraxetin triggered mitochondrial apoptosis by blocking the MAPK and Akt signaling pathways (Ham et al., 2024). However, fraxetin is a high-risk PAINS compound; these findings require cautious interpretation. There is limited research on the pharmacokinetics, bioavailability, and long-term safety of fraxetin.

Baicalein is a trihydroxyflavone derived from *Scutellaria baicalensis* Georgi [Lamiaceae]. In an EMs mouse model, administration of 40 mg/kg baicalein for 28 days reduced endometrial lesions and inflammatory cytokines. *In vitro*, treatment of immortalized human ovarian endometriotic stromal cells (ihOESCs) with 0, 1, 2, and 5 μg/mL baicalein reduced ihOESC viability and increased Bax, BAK, and caspase-1 expression. Baicalein also reduced the levels of antioxidant enzymes GPX1 and SOD1 and decreased phosphorylation of MAPK, JNK, ERK1/2, P70S6K, and S6. In addition, it induced ROS production, consistent with MAPK/PI3K pathway suppression (Park et al., 2024). However, baicalein is a high-risk PAINS compound; these findings require cautious interpretation. Baicalein has poor water permeability, which limits its clinical application.

#### 4.1.2 Targeting the MAPK signaling pathway

The MAPK pathway regulates cell proliferation and apoptosis. The main members include ERK1/2, JNK, and p38MAPK. The ERK pathway exerts anti-apoptotic effects by inhibiting Bim and upregulating Bcl-2 expression, whereas the JNK/p38 pathway initiates apoptosis by activating Bim, Bad, and Bax. Intervention with natural compounds to activate the JNK/p38 pathway or inhibit ERK can re-establish the balance between pro-apoptotic and anti-apoptotic signals. This reverses the resistance to apoptosis in ectopic endometrial cells, thereby preventing EMs progression.

Ursolic acid is a natural compound found in *Rosmarinus officinalis* L. [Lamiaceae]. When HEESCs were treated with 15–60 μM ursolic acid, it inhibited cell viability in a dose-dependent manner, increased caspase-3, p-JNK/t-JNK expression, and reduced VEGF levels. These suggest that ursolic acid inhibited cell proliferation and angiogenesis, and induced apoptosis by modulating p38 and JNK (Li et al., 2020a). However, this study is only done in the laboratory and has not yet been verified in animals or humans, so its *in vivo* efficacy, pharmacokinetics, and toxicity remain unclear.

Silibinin is a flavonolignan obtained from *Silybum marianum* (L.) Gaertn. [Asteraceae]. A 28-day continuous administration of 50 mg/kg silibinin in EMs rats reduced ectopic lesions size, decreased Bcl-2 expression, elevated ERK1/2 expression, increased apoptosis, and lowered angiogenesis, indicating that silibinin alleviated EMs by modulating the ERK1/2 signaling pathway (Nahari and Razi, 2018). *In vivo* study found that intraperitoneal injection of 100 mg/kg silibinin in a surgical EMs mouse model, combined with *in vitro* treatment of VK2/E6E7 and End1/E6E7 cells with 0–50 μM silibinin, demonstrated that silibinin dose-dependently inhibited cell proliferation, suppressed DNA replication, induced ROS production by modulating the MAPK signaling pathway, thereby promoting apoptosis (Ham et al., 2019). However, silibinin is associated with a medium-risk PAINS alert, and its experimental results require cautious interpretation. The long-term toxicity of silibinin and its effects on healthy tissues remain uncertain, limiting its clinical applicability.

Naringenin is a naturally occurring dihydroflavonoid compound. Naringenin at concentrations of 0.5–100 μM inhibited VK2/E6E7 and End1/E6E7 cell growth while promoting apoptosis. Naringenin induced apoptosis through the mitochondrial pathway, causing DNA damage, increasing ROS production, and triggering ER stress. It also increased pro-apoptotic proteins Bax and Bak, reduced Bcl-2 expression, phosphorylated Akt, ERK1/2, and P90RSK, and increased phosphorylated JNK and p38 (Park et al., 2017). However, this study uses a simplified model of immortal cell lines that lacks the key pathological environment of EMs. All experiments were completed within 48 h without long-term treatment, precluding assessment of sustained effects and toxic side effects. Furthermore, the lack of animal studies limits its clinical translation.

Luteolin is a natural flavonoid found in many plants. Luteolin (40 mg/kg) reduced ectopic lesions in EMs mice. In addition, luteolin (20 μM) induced apoptosis by promoting ROS production and enhancing lipid peroxidation. It reduced ERK1/2, JNK, and PI3K/Akt, and increased p38 levels, thereby producing anti-proliferation and pro-apoptosis effects in VK2/E6E7 and End1/E6E7 cells (Park et al., 2019c). However, luteolin is a high-risk PAINS compound; these findings require cautious interpretation. Issues regarding its bioavailability remain unresolved.

Delphinidin is a common flavonoid found in *Solanum melongena* L. [Solanaceae]. 0, 20, 50, and 100 μM delphinidin inhibited VK2/E6E7 and End1/E6E7 growth and induced apoptosis in a dose-dependent manner. It achieved this effect by regulating the cell cycle while down-regulating ERK1/2 and Akt, and upregulating p38MAPK levels (Park et al., 2019b). However, the concentration used in the experiment was high (100 µM), and its safety remains to be verified. This study uses a simplified model of immortal cell lines that lacks the key pathological environment of EMs. Moreover, delphinidin is a high-risk PAINS compound; these findings require cautious interpretation.

Myricetin is a naturally occurring flavonoid. In an EMs mouse model, 30 mg/kg myricetin for 28 days reduced Ccne1 (a biomarker of cell cycle arrest) and inhibited EMs lesion growth. At concentrations of 0–100 μM, myricetin inhibited VK2/E6E7 and End1/E6E7 cell growth, induced ROS production, ultimately leading to apoptosis. This effect was realized through reduced Akt and ERK1/2 and enhanced p38 (Park et al., 2020). These suggest that it may involve the mitochondrial pathway. However, myricetin is a high-risk PAINS compound; these findings require cautious interpretation. Furthermore, there is little clinical research on myricetin.

#### 4.1.3 Targeting the NF-κB signaling pathway

The NF-κB signaling pathway acts as an important bridge between inflammatory responses and cell survival. It affects inflammatory mediators, immune cell development, and apoptosis through its canonical and non-canonical pathways. Targeting this pathway to induce apoptosis has become a key method for treating EMs.

Baicalein is a product of flavonoids. In the laboratory, HEESC treatment with 40 μM baicalein for 48 h reduced cell survival and Bcl-2 expression and blocked the cell cycle. These findings indicate baicalein induced apoptosis by inhibiting the NF-κB pathway and reducing Bcl-2 (Jin et al., 2017). However, the study used cells from normal tissue, limiting the relevance of EMs. Furthermore, baicalein is a high-risk PAINS compound, and these findings need cautious interpretation.

Curcumin is a polyphenol extracted from *Curcuma longa* L. [Zingiberaceae]. Cao et al. found that 48 mg/kg curcumin inhibited NF-κB in mice. It increased Bax/Bcl-2 and caspase-9 expression, thereby promoting apoptosis (Cao H. et al., 2017). However, curcumin is a high-risk PAINS compound; these findings require cautious interpretation. Moreover, curcumin’s low bioavailability in the human body limits its therapeutic potential.

Ginsenoside Rg3 is a saponin compound. Huang et al. found that 100 μg/mL and 150 μg/mL ginsenoside Rg3 reduced HEESC activity, downregulated NF-κB and VEGF expression, and increased caspase-3 levels. These results suggest that ginsenoside Rg3 may inhibit angiogenesis and promote apoptosis by NF-κB pathway suppression (Huang et al., 2020). However, this study lacks animal studies to validate efficacy and safety.

Dehydrocostus lactone is a sesquiterpene lactone extracted from *Aucklandia lappa* Decne. [Asteraceae]. 5, 10, and 20 μM dehydrocostus lactone reduced human EM-12Z cell growth and induced apoptosis. It increased caspase-3/8/9 levels, while decreasing Bcl-2 and IL-10, and it may be caused by NF-κB pathway suppression (Woo et al., 2019). Furthermore, the drug’s effects on pain factors and macrophage polarisation at non-cytotoxic low concentrations (0.5–2 µM) represent a key highlight of this study. However, this study is limited by its reliance solely on *in vitro* experiments using a single endometriotic cell line (12Z). This model lacks the essential pathological microenvironment of EMs (immune, extracellular matrix, and chronic inflammatory contexts). Consequently, the findings are only preliminary and may not translate to the complex human disease. Furthermore, the absence of *in vivo* validation leaves the compound’s overall efficacy in inhibiting lesion growth and alleviating pain entirely unassessed.

Flavokawain A is a small molecule natural substance belonging to the aromatic chalcone family and is extracted from the roots of the *Piper methysticum* G.Forst. [Piperaceae]. After Wei et al. injected 50 mg/kg of Flavokawain A into mouse models with EMs, which minimized EMs lesions and reduced adhesion scores. It reduced NF-κB, PI3K, and Bcl-2, while increasing Bax (Wei et al., 2024). However, flavokawain A is a high-risk PAINS compound; these findings require cautious interpretation.

#### 4.1.4 Targeting the JAK/STAT signaling pathway

The JAK/STAT pathway, controlled by various inflammatory and growth factors, is important to immune-inflammatory responses. In EMs, the JAK/STAT pathway regulates apoptosis. Elevated IL-6 levels in EMs patients activate the JAK/STAT pathway and phosphorylate STAT3. This, in turn, activates Bcl-2, leading to ectopic endometrial cell resistance to apoptosis and proliferation. Therefore, inhibiting the JAK/STAT pathway is of great significance for treating EMs.

Curcumin is an active ingredient extracted from the medicinal plant *Curcuma zedoaria* (Christm.) Roscoe [Zingiberaceae]. In the *in vitro* experiment, EESCs were treated with 5–40 μg/mL curcumin for 48 h. These cells’ vitality and migration deteriorated, and more cells underwent apoptosis. The treatment elevated Bax and caspase-3 while decreasing Bcl-2, TNF-α, and IL-1β levels, and inhibited JAK2 and STAT3, thereby promoting apoptosis. *In vivo*, EMs model rats administered 20 mg/kg curcumin for 28 days showed significantly smaller ectopic lesions. Curcumin also inhibited JAK2, STAT3, and Bcl-2 activation while increasing Bax and caspase-3 (Wang et al., 2022). However, because the study did not assess the effects on NESCs, its superiority over NESCs remains unconfirmed.

#### 4.1.5 Targeting the TGF-β signaling pathway

The TGF-β signaling pathway is an evolutionarily conserved cellular transduction mechanism that precisely regulates apoptosis. Modulation of this pathway is highly significant for EMs treatment.

β-Sitosterol, a tetracyclic triterpenoid, is a naturally occurring bioactive compound. *In vivo* administration of 35 and 350 μg/kg β-sitosterol for 21 days in EMs mice and *in vitro* treatment of EESCs with 30–90 μM β-sitosterol for 48 h inhibited cell growth and migration, decreased TGF-β1 levels, elevated Smad7 expression, and promoted apoptosis. These findings indicate that β-sitosterol exerted therapeutic effects against EMs by inhibiting TGF-β-induced Smad phosphorylation (Wen et al., 2023). However, the transfection efficiency of si-Smad7 *in vitro* experiments was not quantified, potentially influencing the reliability of the results.

Tanshinone IIA, a major bioactive compound of *Salvia miltiorrhiza* Bunge [Lamiaceae]. In an EMs rat model, 30 mg/kg Tanshinone IIA reduced ectopic lesion volume, inhibited epithelial proliferation and angiogenesis, and downregulated TGF-β1, p-SMAD2, and Bcl-2, while increasing Bax and caspase-9. These results suggest that Tanshinone IIA promoted apoptosis by modulating the TGF-β/SMAD (Ma et al., 2021). However, given injection may lower long-term adherence, further studies are needed to optimize dosing regimens.

Picroside II is an active compound from *Neopicrorhiza scrophulariiflora* (Pennell) D.Y.Hong [Plantaginaceae]. Li et al. found that 10 mg/kg and 30 mg/kg picroside II increased Bax levels, reduced Bcl-2, and TGF-β. These findings suggest that picroside II promoted apoptosis by suppressing TGF-β (Li et al., 2020b). However, the study did not provide oral bioavailability data for picroside II.

#### 4.1.6 Targeting the HIF-1α signaling pathway

The HIF-1α pathway is the key mechanism of cell adaptation to hypoxia, which plays an important role in apoptosis. Natural products targeting HIF-1α may provide a promising treatment for EMs.

Rutin is a flavonol with antioxidant effects. In an EMs rat model, rutin at 3 and 6 mg/kg increased Bax, caspase-9, GPX, and SOD levels, while decreasing Bcl-2, p-mTOR, and MDA. TUNEL staining showed elevated apoptosis rates (Talebi et al., 2021). Similarly, in labor-cultured EESCs, treatment with 70 μM rutin reduced cell viability, clonogenicity, migration, and invasion, while enhancing apoptosis. ROS, NOX4, HIF-1α, Bcl-2, Ki-67, MMP2 and MMP9 all decreased, while Bax and caspase-9 increased. These indicate that rutin can suppress NOX4 and block the ROS/HIF-1α pathway, thereby inhibiting EESC proliferation and migration and promoting apoptosis (Wang et al., 2023). However, rutin is a high-risk PAINS compound; these findings require cautious interpretation.

Pro-EGCG is a stable derivative of EGCG with higher bioavailability. Both animal and cell studies demonstrated that pro-EGCG inhibited angiogenesis by suppressing the HIF-1α/VEGF pathway. Pro-EGCG suppressed functional blood vessels and microvessel growth within the lesion, inhibited VEGF production, and promoted apoptosis. Moreover, it exhibited superior bioavailability, antioxidant capacity, and anti-angiogenic activity compared to EGCG (Hung et al., 2024). However, we need to conduct more clinical studies to verify its therapeutic effects and elucidate its mechanisms of action.

#### 4.1.7 Targeting the Bcl-2 signaling pathway

In the Bcl-2 protein family, certain members promote cell survival, while others induce apoptosis. It serves as the core molecular system governing the mitochondrial apoptosis pathways. Natural compounds may suppress EMs progression by inducing apoptosis through Bcl-2 downregulation.

Resveratrol is a non-flavonoid polyphenol that exists in many plants and fruits. Chen et al. found that when EMs rats were administered 15 or 45 mg/kg resveratrol for 28 days, the Bcl-2 levels were downregulated. Resveratrol at 40 μM and 100 μM inhibited the growth and invasion of HEESCs, increased Bax and caspase-3 expression, and induced apoptosis (Chen Z. et al., 2021). However, the tissue distribution of resveratrol varies among different animal models. Therefore, further animal studies are needed to assess its pharmacokinetics and tissue distribution. Moreover, resveratrol is associated with a medium-risk PAINS alert, and its experimental results require cautious interpretation.

Isoliquiritigenin is a natural flavonoid derived from *Glycyrrhiza uralensis* Fisch. ex DC. [Fabaceae]. In an EMs Balb/c mouse model, isoliquiritigenin was administered at 1 or 5 mg/kg for 28 days. The treatment reduced the mass of endometriotic lesions, suppressed inflammatory cytokine elevation, enhanced Bax and caspase-3 expression, and significantly decreased Bcl-2 levels, thereby inhibiting EMs progression and inducing lesion apoptosis. *In vitro*, End1/E6E7 cells treated with isoliquiritigenin (10–100 μM) exhibited marked suppression of proliferation and migration, and increased apoptosis (Hsu et al., 2020). However, isoliquiritigenin is a high-risk PAINS compound; these findings require cautious interpretation. Long-term toxicity studies and clinical trials are needed.

Shikonin is a naphthoquinone compound with antiviral and immunomodulatory properties. In an EMs rat model, shikonin was administered at low (300 mg/kg), medium (500 mg/kg), and high (800 mg/kg) doses for 28 days. Findings indicated that as the dose escalated, the number of transplanted endometrial glands became fewer and the glandular epithelial cells became messy; at the same time, Bax levels increased, Bcl-2 expression decreased, and apoptosis was triggered (Zhang and Tang, 2018). However, shikonin is a high-risk PAINS compound; these findings require cautious interpretation. Safety, efficacy, and pharmacokinetic studies are absent.

Ligustrazine is an active ingredient derived from *Ligusticum striatum* DC. [Apiaceae]. Ligustrazine of 12, 24, and 48 μM inhibited EESC proliferation, upregulated Bax expression, downregulated Bcl-2 levels, and thereby triggered apoptosis. Additionally, it downregulated N-cadherin, upregulated E-cadherin, and consequently suppressed cell invasion (Tang et al., 2023). However, the research was conducted in the laboratory, and the efficacy, safety, and bioavailability of ligustrazine remain unknown.

Betulinic acid is a triterpenoid found in many natural plants. Xiang et al. tested it on human endometriotic epithelial cells (HEECs) and human endometriotic 12Z cells, using 20 μM for 24 h. The treatment reduced the expression of Bcl-2, SOD2, COX2, and MMP1, while increasing caspase-3 expression. It also triggered DNA damage, produced ROS, and impaired mitochondrial function. In particular, betulinic acid reduced IL-6 and TNF-α levels, inhibited cell growth, and promoted apoptosis (Xiang et al., 2020). However, this study lacked *in vivo* experiments and clinical trials.

Naringenin is a flavonoid compound. In the *in vivo* study, naringenin was administered at 50 mg/kg for 21 days in an EMs rat model. The treatment effectively minimised the size and mass of endometriotic lesions, histopathological scores, and serum TNF-α levels. In the *in vitro* experiment, HESCs were exposed to 0.5, 1, and 5 µM naringenin. The treatment inhibited cell growth, downregulated Bcl-2 expression, and upregulated caspase-3 levels, thereby inducing mitochondrial apoptosis to ameliorate EMs (Kapoor et al., 2019). However, the oral bioavailability of naringenin in humans is generally low.

### 4.2 Natural products regulating autophagy for EMs

Recent studies indicate autophagy plays a key role in EMs progression. Natural compounds can modulate autophagy by modulating PI3K/Akt/mTOR and Beclin-1, thereby preventing the occurrence and development of EMs. The natural compounds discussed below will provide us with a scientific basis.

#### 4.2.1 Targeting the PI3K/Akt/mTOR signaling pathway

The PI3K/Akt/mTOR pathway is a negative regulator of autophagy. When inhibited, autophagy will start. mTOR is the key regulator of this pathway. It can be influenced by inflammatory mediators, energy metabolism, and oxidative stress.

Polyphyllin I is a steroid saponin naturally occurring in *Paris polyphylla* Sm. [Melanthiaceae], a species belonging to the Melanthiaceae family. Polyphyllin I inhibited the growth and migration of EESCs and increased Beclin-1, LC3-II, and Bax levels, but reduced Bcl-2, p-Akt, and p-mTOR. It triggered autophagy (Li et al., 2025). The study further links the upstream regulation of autophagy to the classical Akt/mTOR signalling pathway. Nonetheless, this study lacked animal verification or pharmacokinetic evaluation, so its effectiveness and safety remain to be confirmed.

Carvacrol is a phenolic monoterpenoid compound. In *in vivo* experiments, 100 mg/kg carvacrol inhibited endometriotic lesion growth and modulated immune cells. Jang et al. treated ihOESCs with 200 µM carvacrol for 48 h. It was found that carvacrol can reduce calcium ion levels, prevent Akt phosphorylation, and trigger autophagy. This indicates that it may inhibit the PI3K pathway (Jang et al., 2025). However, the stability and safety of this compound, and whether it can really be used in patients, need further research to confirm. Moreover, the animal study had a limited sample size (n = 4). Carvacrol is associated with a medium-risk PAINS alert, and its experimental results require cautious interpretation.

Saikosaponin A is a triterpene saponin extracted from *Bupleurum chinense* DC. [Apiaceae]. Zhou treated HEESCs with 10 μM saikosaponin A and found that it inhibited HEESC growth and migration, increased Beclin-1 and LC3-II levels, and decreased p-Akt/Akt and p-mTOR/mTOR. This component simultaneously triggered apoptosis and autophagy (Zhou, 2024). However, the study lacks animal experiments and needs to assess its *in vivo* efficacy and safety.

Timosaponin AIII is an active ingredient extracted from *Anemarrhena asphodeloides* Bunge [Asparagaceae]. At 7.5 μM, Timosaponin AIII inhibited the proliferation of HEESCs, increased Bax and LC3 levels, and reduced Bcl-2 and p-Akt/Akt. It triggered apoptosis and autophagy (Wan, 2024). However, Timosaponin AIII lacks *in vivo* experimental research and toxicity assessments.

Alpinumisoflavone is a natural isoflavonoid derived from *Erythrina lysistemon* Hutch. [Fabaceae]. 0, 20, and 50 μM Alpinumisoflavone inhibited End1/E6E7 and VK2/E6E7 proliferation, suppressed PI3K/Akt signaling, promoted MAPK phosphorylation, increased Beclin-1 and ATG5 expression, and triggered ER stress and autophagy (Song et al., 2023). However, the study lacked a positive control and used a simplified model of immortal cell lines that lacks the key pathological environment of EMs. Furthermore, Alpinumisoflavone is a high-risk PAINS compound; these findings require cautious interpretation. Alpinumisoflavone lacks *in vivo* experimental research, pharmacokinetic, and toxicity assessments.

#### 4.2.2 Targeting the Beclin-1 signaling pathway

Beclin-1 is the core protein involved in autophagy. Upregulation of Beclin-1 expression can induce autophagy, negatively regulate inflammatory responses, and thereby alleviate the progression of EMs. Research indicates that natural compounds can inhibit EMs progression by regulating the autophagy-related protein Beclin-1.

Berberine is an extract derived from *Coptis chinensis* Franch. [Ranunculaceae] and *Phellodendron amurense* Rupr. [Rutaceae]. When bovine endometrial epithelial cells were treated with berberine at 5, 10, or 20 μM, Nrf2 signaling was activated, Bax/Bcl-2, Beclin-1, and LC3II increased, whereas p62 decreased. This process promoted apoptosis and induced autophagy (Fu et al., 2023). However, this study uses a bovine cell model, and berberine has a moderate risk PAINS alert; the experimental results should be treated with caution. Moreover, berberine’s oral bioavailability is low.

Quercetin is a naturally occurring flavonoid. 15 mg/kg quercetin for 30 days in an EMs rat model increased Nrf2 and Beclin-1, and induced autophagy (Jamali et al., 2021). However, quercetin is a high-risk PAINS compound; these findings require cautious interpretation. Moreover, the issue of its low bioavailability remains to be addressed.

Protopanaxadiol (PPD) is a primary active component extracted from *Panax ginseng* C.A.Mey. [Araliaceae]. It inhibited lesion expansion *in vivo* and suppressed EESC proliferation *in vitro*. It upregulated Beclin-1, Bax/Bak, LC3-II, and PRα, while downregulating Bcl-2/Bcl-xL, Ki-67, and ERα. It also reduced IL-10 expression in NK cells and induced autophagy (Zhang et al., 2018). The low oral bioavailability of PPD hinders its clinical translation.

Gamma Oryzanol is widely present in rice bran and germ. In an EMs rat model, gamma oryzanol was administered at 6 mg/kg. The treatment elevated the levels of Beclin-1 and LC3II, induced autophagy, and thereby prevented EMs development (Eisalou and Farahpour, 2022). However, it is associated with a medium-risk PAINS alert, and its experimental results require cautious interpretation. Moreover, this study did not report pharmacological toxicity, which is crucial for clinical translation.

Soy isoflavones are naturally occurring plant compounds derived from *Glycine max* (L.) Merr. [Fabaceae]. 50 mg/kg soy isoflavones elevated Beclin-1, LC3, and Bax in endometrial tissue of the EMs rat model, decreased Bcl-2 expression, and induced autophagy and apoptosis (Sabetian et al., 2024). However, this study lacks investigation into the effects of other drug concentrations on this model and preclinical pharmacokinetic and long-term toxicity assessments. Moreover, it is associated with a medium-risk PAINS alert, and its experimental results require cautious interpretation.

SCM-198 is an alkaloid derived from *Leonurus japonicus* Houtt. [Lamiaceae]. In an EMs mouse model, SCM-198 was administered at 7.5 and 15 mg/kg. The treatment inhibited ectopic lesion growth, suppressed Bcl-2 and ER levels, promoted Bax, LC3B-II/I, Beclin-1, and PR expression. Laboratory tests found that SCM-198 promoted autophagy-driven apoptosis in EESCs by decreasing ERα and increasing PR expression (Lin et al., 2022). However, it is associated with a medium-risk PAINS alert, and its experimental results require cautious interpretation. Moreover, the study lacks long-term side effects.

Paeonol is a key component derived from *Paeonia suffruticosa* Andrews [Paeoniaceae]. In an EMs rat model, 50 mg/kg paeonol reduced endometrial thickness, decreased inflammatory indicators, and inhibited HIF-1α and Beclin-1. 0–100 μM paeonol inhibited EESC survival in a dose-dependent manner. Paeonol inhibited autophagy by inhibiting HIF-1α, LC3-II, and Beclin-1 (Pang et al., 2021). However, it is a medium-risk PAINS, and the results require cautious interpretation.

### 4.3 Natural products regulating ferroptosis for EMs

Studies indicate that ferroptosis plays a critical role in EMs pathogenesis, presenting a promising therapeutic target. Natural products can effectively interfere with EMs through modulating ferroptosis, showing great potential as therapeutic agents.

#### 4.3.1 Targeting the GPX4 signaling pathway

GPX4 is a phospholipid peroxidase that can suppress lipid peroxides through its antioxidant activity, and it is an important indicator of whether cells will develop ferroptosis. If GPX4 is inhibited, the body’s antioxidant capacity will weaken, leading to the accumulation of ROS and lipid peroxides, which will cause ferroptosis. The following natural compounds have the potential to treat EMs by targeting GPX4.

β-elemene is a sesquiterpene compound derived from *Curcuma wenyujin* Y.H. Chen and C. Ling [Zingiberaceae], which exhibits potent antitumor activity and potential to induce ferroptosis. Through network pharmacology, molecular docking, and bioinformatics analyses, Fu et al. found that β-elemene may regulate ferroptosis in EMs by affecting GPX4, STAT3, and MAPK14. *In vitro*, they treated human endometriotic 12Z cells with 70 μg/mL β-elemene and found that it reduced mitochondrial number, caused cell shrinkage, and increased membrane density. At the same time, p-STAT3, p-MAPK14, GPX4, and GSH levels decreased, while ROS and iron accumulation increased. These changes ultimately inhibited cell growth and migration and triggered ferroptosis. *In vivo*, administration of an EMs mouse model 25, 50, and 75 mg/kg β-elemene for 14 days significantly reduced ectopic lesions and suppressed fibrosis. At the same time, iron deposition increased, and p-STAT3, p-MAPK14, GPX4, and GSH expression decreased. These suggest that β-elemene induces ferroptosis by inhibiting STAT3 and MAPK14, downregulating GPX4 (Fu et al., 2025). However, the current data about pharmacokinetics, safety, and efficacy are limited.

Resveratrol, a polyphenol compound, is abundant in nature. *In vitro*, 100 μM resveratrol inhibited EESC proliferation and migration and caused apoptosis. *In vivo*, 25 mg/kg resveratrol reduced cell numbers in cystic tissues. Whether *in vitro* or *in vivo*, resveratrol reduced GPX4 and GSH expression, while increasing iron, MDA, and ROS levels, eventually triggering ferroptosis, which may be linked to the p53 signaling pathway activation (Zou et al., 2024). However, resveratrol is associated with a medium-risk PAINS alert, and its experimental results require cautious interpretation.

Ginsenoside Rf is a naturally occurring compound. *In vivo*, administration of 3 mg/kg ginsenoside Rf reduced endometriotic lesions. *In vitro,* treatment with 20 µM ginsenoside Rf inhibited EESC growth. Whether *in vivo* or *in vitro*, treatments promoted apoptosis, increased Beclin-1 and LC3-II/I, and decreased GPX4 and SLC7A11 levels. This suggests that ginsenoside Rf helps treat EMs by initiating autophagy-dependent ferroptosis (Zhang L. et al., 2024). However, the study did not examine the pharmacological properties, toxicity, or clinical potential of ginsenoside Rf. More research is needed to prove its safety and effectiveness.

Baicalein is a trihydroxyflavone sourced from *Scutellaria baicalensis* Georgi [Lamiaceae]. The monocyte cell line THP1 was pre-treated to differentiate macrophages. Following pretreatment of THP1 cells with chocolate-like cystic fluid from ovarian endometriotic cysts, FeSO_4_, or a ferroptosis inducer, treatment with 20 μM baicalein suppressed MDA and lipid peroxides, increased GPX4, and restored ferroptosis-mediated phagocytic function in macrophages or induced apoptosis *in vitro* (Yi et al., 2022). However, findings are derived from a macrophage model, which is less relevant to EMs. Furthermore, baicalein is a high-risk PAINS compound; these findings require cautious interpretation.

#### 4.3.2 Targeting the Nrf2 signaling pathway

Nrf2 participates in iron metabolism regulation, GSH synthesis, and NADPH regeneration, and its upregulation inhibits ferroptosis. Moreover, Nrf2 promotes the expression of GPX4, which enhances cellular antioxidant capacity and reduces lipid peroxidation. By modulating the Nrf2 signaling pathway, natural products can regulate redox reactions in cells and prevent damage caused by inflammation and oxidative stress, thus controlling ferrotosis, which is highly promising for treating EMs (Chen et al., 2017).

Pachyic acid is extracted from *Wolfiporia cocos* (Schwein.) Ryvarden & Gilb. [Polyporaceae]. In EMs rats, 3.5 and 7 mg/kg pachymic acid reduced TNF-α and IL-6. *In vitro*, 40 and 80 mg/L pachymic acid reduced EESC growth and increased apoptosis. Both showed that pachymic acid activated the AMPK/Nrf2 pathway. These effects reduced inflammation and iron accumulation and suppressed ferroptosis (Li et al., 2024). However, pachymic acid’s poor solubility limits its effectiveness and clinical application.

Wogonin is a natural hydroxyflavone extracted from *Scutellaria baicalensis* Georgi [Lamiaceae]. 7 mg/kg and 14 mg/kg wogonin reduced IL-1β, IL-6, and iron levels, while increasing Nrf2, GPX, and SLC7A11. These findings suggest that wogonin inhibited ferroptosis in the EMs rat model by activating the SIRT1/Nrf2 pathway (Hu et al., 2023). Future research should focus on its toxicology and stability.

### 4.4 Natural products regulating pyroptosis for EMs

NLRP3 and GSDMD play crucial roles in pyroptosis. When the NLRP3 is activated, it promotes the production of inflammatory factors and cleaves GSDMD, leading to pyroptosis. Therefore, natural products targeting NLRP3 and GSDMD are highly significant for treating EMs.

Fisetin is a natural polyphenol. Arangia et al. found that 40 mg/kg fisetin reduced ectopic lesion growth. Fisetin promoted apoptosis by downregulating Bcl-2 while increasing Bax and caspase-3. Concurrently, it inhibited the NF-κB/NLRP3 signalling. This reduced NLRP3, caspase-1, and reduced inflammatory mediators. These indicate that fisetin exerted therapeutic effects on EMs by promoting apoptosis and inhibiting pyroptosis (Arangia et al., 2023). However, this study lacked a positive control group, and pharmacokinetic and toxic investigations are required. Fisetin is a high-risk PAINS compound; these findings require cautious interpretation.

Tetramethylpyrazine is an amide alkaloid sourced from the traditional Chinese medicine *Ligusticum striatum* DC. [Apiaceae]. In an EMs rat model, tetramethylpyrazine was administered at 19 and 38 mg/kg. It reduced the size of ectopic lesions and also reduced NLRP3, caspase-1, GSDMD-N, and inflammatory cytokines. It even relieved excessive oxidative stress and inhibited NLRP3 inflammasome activation and pyroptosis (Xu et al., 2025). Lacking human data to substantiate the efficacy and safety of Tetramethylpyrazine.

CHS-Iva is a natural substance extracted from *Panax japonicus* (T.Nees) C.A.Mey. [Araliaceae]. In an EMs mouse model, CHS-Iva was administered at 50 mg/kg. The treatment inhibited ectopic lesions’ growth, reduced indicators levels associated with pyroptosis (NLRP3 and GSDMD) and pro-inflammatory substances, thereby reducing pain associated with the disease. Similarly, 10 μM CHS-Iva inhibited EESC pyroptosis and inflammatory responses. These effects are linked to IGF1R/PI3K pathway modulation (Huang Y. et al., 2024). The study’s small sample size, lack of dose groups, and absence of quantitative behavioural pain assessments undermine the reliability of the data. Moreover, CHS-Iva is a high-risk PAINS compound; these findings require cautious interpretation.

Curcumin is a natural polyphenol. When Ding et al. applied it to EESCs at a concentration of 10 μM, the substance showed antioxidant, reducing ROS and MDA, and increasing SOD activity. Moreover, it inhibited pyroptosis by reducing NLRP3, caspase-1, GSDMD, and inflammatory mediators. In an EMs mouse model, curcumin at 300 mg/kg inhibited ectopic lesion growth, reduced ROS levels and inflammatory responses, and suppressed pyroptosis (Ding et al., 2024). However, the sample size is small (n = 5) and curcumin is a high-risk PAINS compound; these findings require cautious interpretation.

Paeonol is an active phenolic compound derived from *Paeonia suffruticosa* Andrews [Paeoniaceae]. In the animal study, 12.5, 25, and 50 mg/kg paeonol minimized ectopic lesions. 50 and 100 μM paeonol inhibited mouse endometriotic epithelial cells (mEECs) growth. In both live mice and cell experiments, paeonol reduced inflammatory factors, downregulated NLRP3, ASC, caspase-1, and GSDMD-N, and increased Nrf2, HO-1, and NQO-1(Du et al., 2024). However, paeonol is a medium-risk PAINSt, and these results require cautious interpretation. Moreover, its bioavailability remains unclear.

### 4.5 Natural products regulating necroptosis for EMs

Although there is currently no basic research on using natural products to specifically trigger necroptosis for treating EMs, this shows that we urgently need to do more in-depth exploration in this regard. The relationship between necroptosis and EMs incidence is particularly complex and important. EMs is an inflammatory condition marked by ectopic endometrial tissue growth. The final step in necroptosis is to release inflammatory mediators and chemokines, which then trigger a violent inflammatory chain reaction—the mechanism is similar to the pathological process of EMs. Moreover, ectopic lesions of EMs form in an inflammatory environment, especially when TNF-α levels are elevated, which makes it particularly easy to initiate necroptosis. Additionally, ectopic endometrial tissues in EMs patients exhibit higher levels of necroptosis mediators, including RIPK3 and MLKL, compared with normal tissues, providing indirect support for necroptosis’s involvement in EMs (Chen and Lu, 2020). Natural products demonstrate remarkable promise in regulating necroptosis. For example, natural products such as baicalin (Huang et al., 2022), tanshinone (Guo et al., 2018), and curcumin (Erfen and Akbay Çetin, 2022) have been reported to influence necroptosis. Related studies have primarily focused on tumors, neurodegenerative diseases, or ischemia-reperfusion injury. Although these natural products have also demonstrated anti-EMs effects, their mechanisms are typically attributed to antioxidant, anti-inflammatory, anti-proliferative, or pro-apoptotic properties. No study has yet thoroughly investigated or confirmed that their efficacy is achieved through precise modulation of the necroptosis pathway. Therefore, in the future, we will systematically explore how natural products can treat EMs by targeting necroptosis. The potential value of this matter is obvious.

## 5 Clinical translation: therapeutic strategies and synergistic potential of natural products

Although extensive preclinical studies have demonstrated the potential of natural products such as curcumin and resveratrol to treat EMs by regulating PCD, validation of their clinical efficacy remains crucial for translation into practice. Current clinical studies, though limited in scale, reveal their possibilities as adjuvant or alternative therapies and show broad prospects for combination with existing standard treatments (Table 3).

**TABLE 3:** Clinical trials of natural products for treating EMs.

Natural product	Study title	Study number	Status	Phase	Control	Dosage regimen	Sample size	Results/Outcome measures
Curcumin	Effect of curcumin on painful symptoms of endometriosis: a triple-blind randomized controlled trial	IRCT20120718010324N66	Completed	Phase 3	Placebo	500 mg, bid, 8 weeks	T:34, C:34	Negative: No significant improvement in pain visual analog scale (VAS) or Endometriosis Health Profile (EHP-30) compared to placebo
Curcumin	Add-on effect of curcumin to dienogest in patients with endometriosis: a randomized, double-blind, controlled trial	IRCT20221111056469N1	Completed	Not Applicable	Placebo + dienogest (2 mg)	80 mg nano-curcumin + dienogest (2 mg), daily, 8 weeks	T:43, C:43	Positive: Superior to dienogest alone in reducing pain, improving quality of life (QOL), and Female Sexual Function Index (FSFI) scores
Curcumin	Flexofytol® for the Treatment of Endometriosis- Associated Pain	NCT04150406	Recruiting	Not Applicable	Placebo	42 mg, bid, 4 months	54 (Planned)	Not Posted Primary: Pain score (Numerical Rating Scale, NRS). Secondary: EHP-30, FSFI.
Resveratrol	The use of resveratrol as an adjuvant treatment of pain in endometriosis: a randomized clinical trial	NCT02475564	Completed	Phase 4	COC + Placebo	COC + resveratrol 40 mg, daily, 42 days	T:22, C:22	Negative: No added pain relief (VAS) over COC alone. CA-125 decreased in both groups with no intergroup difference
Resveratrol	Effects of resveratrol on the expression of inflammatory factors and progesterone receptors in the endometrium of women with endometriosis	IRCT2015101724569N1	Completed	Phase 2	Placebo	400 mg, bid, 12 weeks	T:17, C:17	Positive (Molecular): Downregulated MMP-2, MMP-9, VEGF, and TNF-α expression in tissue
Silymarin	A randomized trial assessing the efficacy of Silymarin on endometrioma-related manifestations	IRCT20150905023897N5	Completed	Phase 2	Placebo	140 mg, bid, 12 weeks	T:35, C:35	Positive: Reduced IL-6, cyst volume, and pain (VAS). No change in EHP-30 or FSFI.
Astaxanthin	Astaxanthin ameliorates inflammation, oxidative stress, and reproductive outcomes in endometriosis patients undergoing assisted reproduction: A randomized, triple-blind, placebo-controlled clinical trial	IRCT20220625055274N1	Completed	Phase 3	Placebo	6 mg, daily, 12 weeks	T:25, C:25	Positive: Improved inflammatory and oxidative stress markers; increased oocyte yield, mature oocytes, and top-quality embryos
Medicinal Cannabis	Challenges in conducting a feasibility RCT of medicinal cannabis for endometriosis pain in Australia	ACTRN12622001560785	Terminated	Phase 2/3	Placebo oil/Cannabidiol oil only	50–300 mg cannabidiol oil + vaporized tetrahydrocannabinol, daily, inhalation, 3 months	63 (Planned)	Terminated: Failed due to recruitment challenges and high dropout. Efficacy not assessed
Cannabidiol	Cannabidiol and management of endometriosis pain	NCT04527003	Completed	Phase 2	Placebo	10/20 mg, daily, 8 weeks	2 (10 mg), 1 (20 mg), 4 (Control)	Positive: Reduced pain (NRS) compared to control
Cannabidiol	Evaluating the impact of a novel cannabinoid product for endometriosis	NCT06477406	Recruiting	Phase 2	Placebo	0.75 mL, tid, 12 weeks	30 (Planned)	Not Posted Primary: Pain VAS. Secondary: EHP-30, Beck Anxiety Inventory (BAI), Beck Depression Inventory (BDI), and inflammatory biomarkers
Cannabidiol	Cannabidiol for the treatment of pelvic pain in endometriosis	NCT05670353	Terminated	Phase 3	Placebo	10–150 mg, daily, 9 weeks	50 (Planned)	Terminated Primary: Pain threshold. Secondary: Anxiety/depression scales, liver/kidney function, adverse events
EGCG	Green tea extract for endometriosis treatment	NCT02832271	Completed	Phase 2	Placebo	400 mg, bid, 3 months	185 (Planned)	Not Posted Primary: Change in lesion size (MRI). Secondary: Pain scores (VAS), quality of life (SF-36), histopathology, angiogenesis metrics (DCE-MRI), and adverse events
Quercetin	Effect of quercetin supplementation on endometriosis outcomes	NCT05983224	Recruiting	Not applicable	Placebo	500 mg, bid, 12 weeks	50 (Planned)	Not Posted Primary: TNF-α, IL-6. Secondary: FBS, HbA1C, Testosterone, Estrogen, FSH, LH, Progesterone, Adiponectin, IGF1, SHBG.

Some clinical trials have not posted their results on public registries (clinicaltrials.gov). Consequently, they are marked as “Not Posted” under the “Results/Outcome Measures” column, while their planned primary and secondary outcome measures have been documented.

### 5.1 Clinical trials of natural products for treating EMs

Gudarzi et al. found that 500 mg curcumin for 8 weeks failed to significantly improve pain or quality of life in EMs patients compared with the placebo group (Gudarzi et al., 2024). Another study showed that nano-curcumin (80 mg) combined with dienogest (2 mg) treatment for 8 weeks reduced pain scores (including dysmenorrhea, dyspareunia, and chronic pelvic pain) and improved quality of life and sexual function in patients with stage II-III EMs (Sargazi-Taghazi et al., 2025). The discrepancy may stem from variations in the bioavailability of curcumin formulations, whether combined with hormonal therapy, and differences in study population characteristics. The latter study used a nanoparticle formulation, whose bioavailability is approximately 50 times higher than that of conventional curcumin, thereby enhancing its biological effects.

Mendes et al. found that a low dose of resveratrol (40 mg) combined with a compound oral contraceptive (COC) for 42 days was not significantly better than placebo in alleviating EMs pain (Mendes da Silva et al., 2017). The low dose and short duration used in this study may affect the effects. In contrast, two other randomized exploratory trials used a higher dose of resveratrol (800 mg) for 12 weeks and found it reduced MMP-2, MMP-9, VEGF, and TNF-α levels in ectopic endometrial tissue (Kodarahmian et al., 2019; Khodarahmian et al., 2021). This suggests that resveratrol may improve EMs by inhibiting lesion invasion, angiogenesis, and inflammation. However, the sample size of these studies is small (34 cases) and only stage III-IV patients are included, so the results may not apply to all patients. In addition, the studies only measured biomarkers and did not evaluate clinical symptom improvement.

Mirzaei et al. conducted a randomized controlled trial to evaluate the effect of silymarin (140 mg, bid, 12 weeks) in combination with dienogest (2 mg). It significantly reduced cyst volume, IL-6 levels, and pain scores, but did not improve quality of life or sexual function (Mirzaei et al., 2022). However, this study’s sample size is small, and the follow-up time is short; it is difficult to assess the long-term effect and recurrence. In addition, the oral bioavailability of silymarin is low. Future research needs larger sample sizes, longer follow-up periods, and formulation technologies to improve bioavailability and verify its efficacy.

Rostami et al. investigated the adjuvant role of astaxanthin in patients with infertility of III/IV EMs (Rostami et al., 2023). Astaxanthin (6 mg) for 12 weeks improved oxidative stress (increased serum total antioxidant capacity and reduced MOD) and inflammatory status (reduced IL-1β, IL-6, TNF-α), and increased the number of oocytes retrieved and the rate of high-quality embryos, but did not significantly improve the final pregnancy rate. However, the study had a small sample size and failed to elucidate pharmacokinetics.

### 5.2 Synergistic effects and mechanistic insights of combination therapy

Given the limitations of monotherapy with natural products for treating EMs and the multifactorial pathogenesis of EMs, combining natural products with existing therapies (hormonal, surgical) is considered a more promising treatment strategy. It not only enables multi-pathway synergistic intervention against the disease but may also reduce recurrence rates and minimize drug side effects. This synergistic effect may stem from multi-target regulation of the disease network, particularly through enhanced modulation of PCD. Hormonal drugs such as dienogest can inhibit estrogen effects and induce apoptosis through the progesterone receptor pathway. Natural products such as curcumin and silymarin can promote apoptosis by regulating the Bcl-2/Bax ratio and activating the caspase cascade, and upregulate the autophagy-related protein LC3-II to activate protective autophagy. When used in combination, natural products can enhance the sensitivity of lesion cells to hormone-induced death signals or initiate PCD, which clinically manifests as more significant pain relief and lesion reduction compared to hormone therapy alone. Natural products (e.g., resveratrol, astaxanthin) have strong antioxidant and lipid metabolism regulatory capabilities, making lesion cells more sensitive to ferroptosis by inhibiting GPX4 or affecting pyroptosis by regulating NLRP3. In postoperative adjuvant therapy, natural products regulate the inflammatory microenvironment and clear residual lesion cells, thereby reducing recurrence.

The synergistic effect of combined therapy is also reflected in the remodeling of the inflammatory microenvironment and immune status of the lesions. The anti-inflammatory (inhibiting NF-κB and COX-2), anti-angiogenic (downregulating VEGF), and anti-invasive (regulating MMPs) effects of natural products can synergize with the hormone pathway inhibition of hormonal therapies, creating a microenvironment unfavorable for ectopic endometrial cells. More importantly, a combination of natural products with gonadotropin-releasing hormone agonists (GnRH-a) may alleviate the latter’s oestrogen-deficiency side effects while preserving therapeutic efficacy. After surgical removal of ectopic lesions, adjuvant use of natural products may reduce recurrence by inhibiting inflammation and angiogenesis in residual lesions.

## 6 Discussion

EMs is a gynecological inflammation that relies on estrogen. The disease arises from endometrial-like tissue growing external to the uterus and bleeding cyclically like menstruation. These processes trigger long-term inflammatory responses, organ adhesions, and persistent fibrosis. Modern medicine has made some progress in treating EMs, such as the use of hormone drugs, surgical removal of lesions, and assisted reproductive technology. Although these methods can help some patients relieve symptoms and improve their fertility chances, there are still many problems, such as frequent recurrence and significant side effects of long-term medication. Therefore, finding new, more effective, and safer treatments has become critical in EMs research.

Many studies show that dysregulation of PCD plays a key role in EMs development. Regulating various PCDs can markedly improve EMs pathology and inhibit ectopic lesion growth. Therefore, this review systematically summarizes the effects of natural products in targeting various PCD forms and their key signaling pathways in EMs. Among them, apoptosis is the most extensively studied form of PCD. Ectopic endometrial cells resist apoptosis through the synergistic dysregulation of dual pathways, and enhancing their apoptosis is key to natural product anti-EMs. Autophagy exerts a dual-function impact on EMs. In the early stages of the disease, autophagy inhibition promotes ectopic implantation, while in the late stages, autophagy activation sustains lesion adaptation and progression. Regulating autophagy activity is a potential strategy for natural products to interfere with EMs. In addition, the roles of other forms of PCD, including ferroptosis, pyroptosis, and necroptosis, are gradually being revealed in EMs, which provides a broad opportunity for new therapeutic compounds.

Notably, various PCDs do not exist alone but interact through a dynamic network (Kopeina et al., 2025). There are several key connection nodes among PCDs. Bcl-2 is a crucial hub regulating apoptosis and autophagy. It can inhibit Bax/Bak on mitochondria to inhibit apoptosis and combine with Beclin-1 to inhibit autophagy, thereby serving as a negative regulator of both processes (Kist and Vucic, 2021). Caspase-3 and caspase-8 can induce apoptosis and cleave the pyroptosis execution protein GSDME, thereby transforming apoptosis into pyroptosis (Zhang W. et al., 2024). Beyond these molecular switches, organelle platforms also play a key role. Mitochondria are critical: their outer membrane permeabilization can induce apoptosis, while they are also the main place of lipid peroxidation in ferroptosis (Wang L. et al., 2023). In addition, mtDNA and oxidized cardiolipin released from damaged mitochondria activate the NLRP3 inflammasome and induce pyroptosis. Mitochondrial autophagy, by clearing damaged mitochondria, reduces ROS to suppress ferroptosis and can also eliminate inflammasome activation to inhibit pyroptosis (Tang et al., 2019). At the metabolic level, ROS, iron ions, and GSH constitute a core metabolic center. Moderate ROS can induce apoptosis, while excessive lipid peroxidation and ROS induce ferroptosis. Iron ions promote ferroptosis through the Fenton reaction and can also influence apoptosis/autophagy-related pathways. Depletion of GSH can inactivate GPX4, leading to ferroptosis, increase cellular sensitivity to apoptosis, and regulate autophagy through the Nrf2 axis. Notably, GSH depletion itself can directly activate the NLRP3 inflammasome, thereby inducing pyroptosis. In addition, lipid peroxides, as a hallmark of ferroptosis, can also directly activate the NLRP3 inflammasome to initiate pyroptosis. The therapeutic advantage of natural products lies in their ability to exert multi-node synergistic intervention on the PCD network, exceeding the efficacy of single-target drugs. For example, in balancing autophagy and apoptosis, SCM-198 enhances protective autophagy by balancing ERα and PR signals. This process involves Bcl-2 and Beclin-1, thereby inhibiting ectopic endometrial cell proliferation (Lin et al., 2022). β-elemene disrupts mitochondrial function through its lipophilicity and induces mitochondrial apoptosis and ferroptosis. The lipid peroxides produced can then act as danger signals to activate the NLRP3 inflammasome, triggering pyroptosis and achieving a multi-targeted effect on the lesion (Fu et al., 2025). Regarding the regulation of the apoptosis-pyroptosis switch, fisetin inhibits NF-κB/NLRP3 inflammation, modulates caspase-1 and caspase-3, and reduces the inflammatory response caused by excessive pyroptosis (Arangia et al., 2023). Ginsenoside Rf increases Beclin-1 and LC3-II/I levels while decreasing SLC7A11 and GPX4 expression, inducing autophagy-dependent ferroptosis to improve EMs (Zhang L. et al., 2024). In summary, natural products can regulate molecules such as Bcl-2 and caspase, stabilize functional platforms such as mitochondria, and interfere with metabolic-inflammatory signals such as ROS, iron, GSH, and lipid peroxides, thus regulating multiple nodes of the PCD network. This multi-target, network-based mechanism of action underlies their synergistic therapeutic effects and their ability to overcome drug resistance.

Recently, natural products have attracted particular attention in PCD for treating EMs because of their wide sources, ability to act on multiple targets, and safety. Their unique therapeutic advantages are closely linked to their specific chemical structures, which fundamentally determine their bioactivity and therapeutic effects. Their main advantages are as follows: (1) With their complex chemical structures, natural products can regulate multiple targets and pathways, intervening in several key pathological processes of EMs at once. The molecular basis of this advantage lies in the diverse pharmacophores within natural product structures, which interact with multiple disease-related targets. For example, the catechol structure in the flavonoid quercetin is a potent redox-modulating center. It can induce oxidative stress by generating ROS, leading to mitochondrial dysfunction, thus activating the intrinsic apoptosis pathway. In addition, the overall planar aromatic structure of quercetin allows it to act as a competitive inhibitor, embedding the ATP-binding pockets of kinases such as PI3K, Akt, and MAPK (Park et al., 2019a). (2) Some natural products can coordinately regulate multiple PCDs, creating networked therapeutic effects. Saponin compounds interact with cell and lysosomal membranes through their amphiphilic structure (hydrophobic aglycone and sugar chain). By interfering with membrane fluidity, affecting membrane receptor function, or altering lysosomal activity, they regulate autophagy. Their sustained oxidative stress depletes GSH and inhibits GPX4, triggering ferroptosis (Zhang L. et al., 2024). The lipophilic polycyclic skeleton of the terpenoid β-elemene allows it to accumulate in mitochondrial membranes, inducing apoptosis by disrupting mitochondrial function. ROS accumulation and metabolic abnormalities can activate MAPK and STAT3, which inhibit SLC7A11 and GPX4, thereby inducing ferroptosis (Fu et al., 2025). (3) Most natural products come from creatures in nature. They are very biocompatible and have lower toxicity and side effects than traditional hormone drugs. The chemical structures of many natural products resemble substances in our bodies. For example, the aglycones of saponins are often steroids or triterpenes, and their structures are very similar to membrane components such as cholesterol. The phenolic hydroxyl groups in flavonoids and polyphenols are common functional groups in the body’s antioxidant network. This structural similarity makes them easier to be recognized and metabolized by biological enzyme systems, reducing the generation of toxic metabolites. Moreover, their multi-target mode of action avoids the severe physiological imbalances that are caused by a single receptor. (4) Some natural products show preventive potential in early disease intervention, making them promising candidates for primary prevention of EMs. Structures such as catechols and resveratrol, which are rich in polyphenols and flavonoids, are efficient antioxidants. In the early stage of EMs, these structures can directly neutralize excess ROS, eliminating free radical signals that promote ectopic endometrial cell adhesion, survival, and angiogenesis. Moreover, these groups inhibit the activation of core pro-inflammatory signaling pathways, curbing the formation of a pro-inflammatory microenvironment and providing novel approaches for primary prevention of EMs. However, the specific chemical structures often lead to pharmaceutical bottlenecks such as poor absorption, rapid metabolism, and low bioavailability. Specifically, the catechol structure in flavonoids and polyphenols induces chemical instability, rapid metabolism, and low bioavailability. The large and polar sugar moieties in saponin compounds severely hinder their intestinal absorption. Terpenoids are usually poorly water-soluble because of their highly lipophilic nature. The planar conjugated cationic structure of alkaloid compounds is positively charged, which limits their passive diffusion and transmembrane absorption. These structurally determined pharmacokinetic defects are critical challenges that must be systematically overcome in the transformation of natural products into ideal drug candidates.

Although it is promising that natural products can affect the development of EMs by regulating PCD, their clinical translation still faces several key challenges: (1) The reliability of evidence is weakened by PAINS interference and flaws in study design. Natural products such as quercetin, baicalein, and curcumin are high-risk PAINS compounds. Their *in vitro* activity may stem from non-specific interference, requiring cautious interpretation of their positive results. Concurrently, design flaws in studies, such as the lack of positive control groups, incomplete dose-response designs, and insufficient comparisons with the efficacy of first-line therapies, further undermine the credibility and translational potential of existing evidence. (2) The clinical predictive value of current experimental models is limited. Most current research uses immortalized cell lines such as VK2/E6E7 or is conducted under highly simplified *in vitro* conditions, making it difficult to imitate the complex inflammatory, immune, and hypoxic microenvironment of human EMs. Commonly used animal models, such as mouse autotransplantation models, can partially simulate lesion attachment and growth but inadequately reproduce core clinical features of EMs, such as chronic pain, progressive fibrosis, and infertility. The therapeutic outcomes obtained from these models have limited clinical predictive value. (3) There are significant gaps in critical preclinical development stages (pharmacokinetics, pharmacodynamics, toxicology). Most natural products have shortcomings in pharmacokinetics, such as poor oral absorption, low bioavailability, and unclear *in vivo* metabolic processes. For example, the phenolic hydroxyl groups in flavonoids lead to rapid and extensive metabolic conversion; the bulky sugar moieties in saponins hinder their intestinal absorption. Moreover, systematic pharmacodynamic and toxicological evaluation data are lacking, obstructing the progression from *in vitro* activity to *in vivo* efficacy and eventual clinical application. (4) There is a severe shortage of high-quality clinical evidence. Current supporting evidence mainly comes from preclinical studies, which are a low level of clinical evidence. There is a lack of large-scale, rigorously designed clinical trials with sufficient sample sizes and long-term follow-up to verify their effectiveness and safety. In addition, the optimal protocols (regarding dosage, timing, and duration) for using natural products alone or in combination with existing therapies have not been determined, and their potential synergistic mechanisms require further elucidation. (5) Insufficient depth and systematicity in mechanism elucidation. Existing research mostly focuses on single pathways or individual PCD forms, lacking systematic analysis of the multi-target, multi-pathway synergistic effects mediated by natural products. This makes it difficult to elucidate their complex, network-based mechanisms of action.

Future research should focus on the following directions: (1) Enhance the rigor and reliability of evidence from the source. For high-risk PAINS compounds, a stricter validation framework must be established, including multiple experimental methods to verify it. Moreover, *in vivo* pharmacodynamic validation must be included as an essential step to verify its activity. In the research design, adding standard first-line therapies as positive controls will improve study credibility and clinical relevance. (2) Optimize and use more pathologically relevant models to improve the predictive value of efficacy findings. (3) Focus on solving pharmacokinetic problems, enhancing the bioavailability and stability of natural products through formulation improvements and structural optimization. (4) Design rigorous randomized controlled clinical trials with large samples and long follow-up. Actively explore combination strategies with existing standard therapies to translate from basic research to clinical benefit. (5) Using multi-omics technologies such as proteomics and metabolomics to systematically analyze the interaction networks among different PCD, providing a basis for developing multi-target therapies. Through implementing these strategies, translating more natural products into clinical drugs, ultimately offering more effective treatment options for EMs patients.

## 7 Conclusion

In summary, this paper offers a detailed review of various mechanisms by which natural products treat EMs by targeting various forms of PCD and its key signaling pathways. Due to their unique advantages, including multi-target action capabilities, multi-pathway regulatory properties, and the ability to coordinate the regulation of PCD networks, natural products have become promising candidate therapies in treating EMs. A large number of studies *in vivo* and animal investigations demonstrate that natural products such as quercetin, luteolin, resveratrol, and curcumin can effectively regulate multiple forms of PCD, including apoptosis, autophagy, ferroptosis, and pyroptosis, regulating key signaling pathways including PI3K/Akt, MAPK, NF-κB, Nrf2, and GPX4, thereby inhibiting the development of EMs.

However, we must be clearly aware that research in this field still faces many difficulties, including a lack of high-quality clinical evidence, limited mechanism research, and unclear pharmacokinetic properties. If we want to transform natural product treatment to EMs from lab research to clinical practice, future studies require urgent interdisciplinary collaboration to systematically resolve critical challenges. In the preclinical stage, PAINS risk assessment must be carefully carried out to avoid futile subjects. During the development phase, bioavailability must be addressed through structure optimization techniques. At the clinical verification stage, well-designed, multicenter randomized controlled trials are needed to confirm their efficacy and safety. Only through such multi-level and cross-disciplinary collaboration can the therapeutic potential of natural products targeted to PCD be fully released, thereby promoting innovation in EMs treatment strategies.

## Glossary

Akt: Protein Kinase B
Bax: BCL2-Associated X protein
Bcl-2: B-cell lymphoma/leukemia-2
DISC: Death-inducing signaling complex
EMs: Endometriosis
ER: Endoplasmic reticulum
ERK: Extracellular signal-regulated kinase
FADD: Fas-associated death domain
GPX4: Glutathione Peroxidase 4
GSDMD: Gasdermin D
GSH: Glutathione
HO-1: Heme Oxygenase-1
IhOESCs: Immortalized human ovarian endometriotic stromal cell lines
IL-6: Interleukin 6
IL-1β: Interleukin-1β
LC3: Light Chain 3
MAPK: Mitogen-activated protein kinase
MDA: Malondialdehyde
MLKL: Mixed lineage kinase domain-like protein
MMP: Matrix metalloproteinase
mTOR: mammalian target of rapamycin
NF-κB: Nuclear Factor kappa-B
NLRP3: NLR family pyrin domain-containing protein 3
Nrf2: Nuclear factor erythroid 2-related factor 2
PCD: Programmed cell death
PI3K: Phosphatidylinositol 3-kinase
RIPK: Receptor-interacting protein kinase
ROS: Reactive oxygen species
SOD: Superoxide dismutase
TGF-β: Transforming Growth Factor Beta Receptor
TNF-α: Tumor necrosis factor-α
HIF-1α: Hypoxia-Inducible Factor -1α
