# Supplementary material for: Natural products modulate programmed cell death signaling mechanism for treating endometriosis: a review
Source: Front Pharmacol. 2026 Jan 29;17:1742212. doi: 10.3389/fphar.2026.1742212 (PMC12894019; doi:10.3389/fphar.2026.1742212)
Supplement: Supplementary file 1 [file Table1.docx]

**Supplementary Table S1.** Detailed experimental parameters, controls, and PAINS risk assessment for the natural products listed in Table 1.

| **Natural products** | **Duration** | **Type of extract** | **Controls** | **Toxic side effects** | **Obstacles to development** | **PAINS risk** | **Evidence level** | **Notes on PAINS assessment** |
| --- | --- | --- | --- | --- | --- | --- | --- | --- |
| Ginsenoside Rg3 | 21 d | Pure compound | Sham operation group; Positive control, gestrinone | Not assessed | Lack of clinical data; Unknown pharmacokinetics | Low risk (saponin class, no known PAINS substructures) | Level 2 *(in vivo* + mechanistic exploration) |  |
| Chrysin | 48 h | Pure compound | Vehicle control | Less effects on normal endometrial cells at lower concentrations; significant reduction in normal cell viability at high concentration (100μM) | Lack of clinical data; Unknown pharmacokinetics; Lack of *in vivo* model validation; Only in immortalized cell lines (oversimplified, lacks disease microenvironment) | Low risk (flavone class, no known PAINS substructures) | Level 3 (verified *in vitro*) |  |
| Apigenin | 48h | Pure compound (purchased from Sigma-Aldrich) | Untreated cells | Not assessed | Lack of clinical data; Unknown pharmacokinetics; Lack of *in vivo* model validation; Potential low bioavailability; Only in immortalized cell lines (oversimplified, lacks disease microenvironment) | Low risk (flavone class, no known PAINS substructures) | Level 3 (verified *in vitro*) |  |
| Quercetin | In vitro: 48 h; In vivo: 4 w | Pure compound (purchased from Sigma-Aldrich) | *In vitro*: Untreated cells; *In vivo*: Sham surgery group | Mentioned lower toxicity to primary eutopic endometrial cells compared to ectopic cell lines, but no systematic evaluation of *in vivo* toxicity or long-term side effects was conducted | Lack of clinical data; Unknown pharmacokinetics; Low oral bioavailability | High risk (flavonol class, contains the catechol_A substructure, a known PAINS motif) | Level 2 *(in vivo* + mechanistic exploration) | High PAINS risk, results require cautious interpretation |
| Fraxetin | In vitro: 48 h; In vivo: 4 w | Pure compound | *In vitro*: Untreated cells; DMSO/Vehicle-treated cells.  *In vivo*: Sham surgery group; Vehicle-treated group; Dienogest-treated group. | *In vitro*: Safe at tested concentrations. *In vivo*: Safe at 30 mg/kg | Lack of clinical data; Unknown pharmacokinetics | High risk (Coumarin class, likely contains the catechol_A substructure, a known PAINS motif) | Level 2 *(in vivo* + mechanistic exploration) | High PAINS risk, results require cautious interpretation |
| Baicalein | In vitro: 48 h; In vivo: 4 w | Pure compound | *In vitro*: Untreated cells; DMSO/Vehicle-treated cells.  *In vivo*: Vehicle-treated group; Dienogest-treated group | *In vitro*: Safe at ≤20 µg/ml; Toxic at ≥50 µg/ml. *In vivo*: Safe at 40 mg/kg | Lack of clinical data; Unknown pharmacokinetics | High risk (Flavone class, contains the catechol_A substructure, a known PAINS motif) | Level 2 *(in vivo* + mechanistic exploration) | High PAINS risk, results require cautious interpretation |
| Ursolic acid | 24 h | Pure compound | Untreated cells | Not assessed | Lack of *in vivo* validation; Lack of clinical data; Unknown pharmacokinetics | Low risk (Triterpenoid class, no known PAINS substructures) | Level 3 (verified *in vitro*) |  |
| Silibinin | In vitro: 48 h; In vivo: 4 w | Pure compound (purchased from Sigma-Aldrich) | *In vitro*: DMSO vehicle-treated cells. *In vivo*: DMSO vehicle-injected mice. | Not assessed | Lack of clinical data; Unknown pharmacokinetics | Medium risk (flavonolignan class, contains polyhydroxy motifs associated with PAINS, requiring verification) | Level 2 *(in vivo* + mechanistic exploration) | Potential PAINS liability, *in vivo* evidence mitigates but does not eliminate risk. |
| Naringenin | 48 h | Pure compound (purchased from Sigma-Aldrich) | Ethanol vehicle control | Not assessed | Lack of *in vivo* validation; Unknown pharmacokinetics; Potential low oral bioavailability; Only in immortalized cell lines (oversimplified, lacks disease microenvironment) | Low risk (flavanone class, no known PAINS substructures) | Level 3 (verified *in vitro*) |  |
| Luteolin | In vitro: 48 h; In vivo: 4 w | Pure compound (purchased from Sigma-Aldrich) | *In vitro*: DMSO vehicle; *In vivo*: DMSO IP injection | Not assessed | Lack of clinical data; Unknown pharmacokinetics | High risk (flavone class, contains the catechol_A substructure, a known PAINS motif) | Level 2 *(in vivo* + mechanistic exploration) | High PAINS risk, results require cautious interpretation |
| Delphinidin | 48 h | Pure compound (purchased from INDOFINE Chemical Company) | DMSO vehicle control | Not assessed | Lack of clinical data; Unknown pharmacokinetics; Lack of *in vivo* model validation; Only in immortalized cell lines (oversimplified, lacks disease microenvironment) | High risk (anthocyanin class, contains a pyrogallol group, a known PAINS feature) | Level 3 (verified *in vitro*) | High PAINS risk, results require cautious interpretation |
| Myricetin | In vitro: 48 h; In vivo: 4 w | Pure compound (purchased from Sigma-Aldrich) | *In vitro*: DMSO vehicle; *In vivo*: DMSO IP injection | Not assessed | Lack of clinical data; Unknown pharmacokinetics; Potential low bioavailability | High risk (Flavonol class, contains a pyrogallol group, a known PAINS feature) | Level 2 *(in vivo* + mechanistic exploration) | High PAINS risk, results require cautious interpretation |
| Baicalein | 48 h | Pure compound (purchased from Sigma-Aldrich/Merck KGaA) | Untreated cells | Not assessed | Lack of clinical data; Unknown pharmacokinetics; Lack of *in vivo* model validation; Only in normal (non-diseased) primary cells (reduced disease relevance) | High risk (Flavone class, contains the catechol_A substructure, a known PAINS motif) | Level 3 (verified *in vitro*) | High PAINS risk, results require cautious interpretation |
| Curcumin | 15 d | Pure compound | Model control | Not assessed | Lack of clinical data; Unknown pharmacokinetics and bioavailability | High risk (Diarylheptanoid class, contains β-diketone/enol and Michael acceptor moieties, known PAINS features) | Level 2 *(in vivo* + mechanistic exploration) | High PAINS risk, results require cautious interpretation |
| Ginsenoside Rg3 | 48 h | Pure compound | Untreated cells | Not assessed | Lack of clinical data; Unknown pharmacokinetics; Lack of *in vivo* model validation | Low risk (saponin class, no known PAINS substructures) | Level 3 (verified *in vitro*) |  |
| Dehydrocostus lactone | 48 h | Pure compound | DMSO vehicle control | Not assessed | Lack of *in vivo* and clinical data; Unknown pharmacokinetics; Only in a single endometriotic cell line (12Z) | Low risk (sesquiterpene lactone class, no known PAINS substructures) | Level 3 (verified *in vitro*) |  |
| Flavokawain A | 6 w | Purified synthetic compound | *In vitro*: Untreated cells; *In vivo*: Model control group, luteolin positive control group | Not assessed | Lack of clinical data; Unknown pharmacokinetics | High risk (Chalcone class, contains a reactive Michael acceptor (α,β-unsaturated ketone), a known PAINS scaffold) | Level 2 *(in vivo* + mechanistic exploration) | High PAINS risk, results require cautious interpretation |
| Curcumin | In vitro: 48 h; In vivo: 4 w | Pure compound | *In vitro*: Untreated cells; AG490 (JAK2 inhibitor, positive control).  *In vivo*: Sham operation group; EMs model group; AG490 (positive control) group | Not assessed | Lack of evaluation on normal endometrial cells; Lack of clinical data; Unknown pharmacokinetics | Low risk (sesquiterpenoid class, no known PAINS substructures) | Level 2 *(in vivo* + mechanistic exploration) |  |
| β-Sitosterol | In vitro: 48 h; In vivo: 3 w | Pure compound | *In vitro*: Untreated cells.  *In vivo*: Model group | Not assessed | Lack of clinical data; Unknown pharmacokinetics | Low risk (phytosterol class, no known PAINS substructures) | Level 2 *(in vivo* + mechanistic exploration) |  |
| Tanshinone IIA | 4 w | Pure compound | Sham operation group; Gestrinone group (positive drug control) | Not assessed | Lack of clinical data; Unknown pharmacokinetics | Low risk (tanshinone class, no known PAINS substructures) | Level 2 *(in vivo* + mechanistic exploration) |  |
| Picroside II | 28 d | Pure compound | Control group (sham operation + saline)； Danazol group (positive drug control) | Not assessed | Lack of clinical data; Unknown pharmacokinetics | Low risk (iridoid glycoside class, no known PAINS substructures) | Level 2 *(in vivo* + mechanistic exploration) |  |
| Rutin | In vitro: 24 h; In vivo: 28 d | Pure compound | *In vitro*: Untreated cells (control group); Ov-NC transfected group (negative control). *In vivo*: Endo-sole group (disease model control); Vitamin C group (positive control) | *In vitro*: Safe at ≤ 70 µM; Toxic at ≥ 90 µM (based on cell viability assay). *In vivo*: Not assessed | Lack of clinical data; Unknown pharmacokinetics; Potentially low oral bioavailability | High risk (Flavonol glycoside class, its aglycone (quercetin) contains the catechol_A substructure, a known PAINS motif) | Level 2 *(in vivo* + mechanistic exploration) | High PAINS risk, results require cautious interpretation |
| Pro-EGCG | In vitro: 48 h; In vivo: 4 w | Purified synthetic compound | *In vitro*: DMSO vehicle control; Positive control (PTK787). *In vivo*: Vehicle control (DMSO in PBS); Positive controls (PTK787, GnRH agonist) | Not assessed | Lack of clinical data; Unknown pharmacokinetics | Low risk (catechin class, no known PAINS substructures) | Level 2 *(in vivo* + mechanistic exploration) |  |
| Resveratrol | In vitro: 48 h; In vivo: 28 d | Pure compound | *In vitro*: DMSO vehicle control. *In vivo*: Sham surgery group; Model control (EMs, vehicle: 0.9% NaCl+35% DMSO) | Not assessed | Lack of clinical data; Unknown pharmacokinetics; Low oral bioavailability | Medium risk (stilbene class, contains polyhydroxy motifs associated with PAINS, requiring verification) | Level 2 *(in vivo* + mechanistic exploration) | Potential PAINS liability, *in vivo* evidence mitigates but does not eliminate risk. |
| Isoliquiritigenin | In vitro: 48 h; In vivo: 28 d | Pure compound | *In vitro*: DMSO vehicle control; Positive control (quercetin). *In vivo*: Sham surgery group; Model control | The article mentions previous studies showing ISL was not toxic to normal endometrial cells at 20 µM, with 76% survival at 50 µM. This study did not assess systemic toxicity of *in vivo* administration. | Lack of clinical data; Unknown pharmacokinetics | High risk (Chalcone class, contains a Michael acceptor and a catechol group, known PAINS features) | Level 2 *(in vivo* + mechanistic exploration) | High PAINS risk, results require cautious interpretation |
| Shikonin | 4 w | Pure compound | Normal rat control group, model + saline group | Not assessed | Lack of clinical data; Unknown pharmacokinetics | High risk (Naphthoquinone class, contains a quinone moiety, may match quinone_A, a known PAINS class) | Level 2 *(in vivo* + mechanistic exploration) | High PAINS risk, results require cautious interpretation |
| Ligustrazine | 48 h | Pure compound | Normal culture control group (no drug, no transfection), Negative control vector group (Experimental high-dose + NC group) | Not assessed | Lack of clinical data; Unknown pharmacokinetics; Lack of *in vivo* validation; Only in ectopic stromal cells (simplified model) | Low risk (pyrazine alkaloid class, no known PAINS substructures) | Level 3 (verified *in vitro*) |  |
| Betulinic acid | 24 h | Pure compound | Empty vector control (CTL), negative control (NC), untreated cell control | Not assessed | Lack of clinical data; Unknown pharmacokinetics; Lack of *in vivo* validation | Low risk (triterpenoid class, no known PAINS substructures) | Level 3 (verified *in vitro*) |  |
| Naringenin | In vitro: 24 h; In vivo: 21 d | Pure compound | *In vitro*: Untreated cells.  *In vivo*: Sham group (no modeling, no treatment), EMs model group (vehicle control after modeling), Dienogest positive drug control group | *In vitro*: Safe at tested concentrations. *In vivo*: Not assessed | Lack of clinical data; Unknown pharmacokinetics; Low oral bioavailability | Low risk (flavanone class, no known PAINS substructures) | Level 2 *(in vivo* + mechanistic exploration) |  |
