# Supplementary material for: Natural products modulate programmed cell death signaling mechanism for treating endometriosis: a review
Source: Front Pharmacol. 2026 Jan 29;17:1742212. doi: 10.3389/fphar.2026.1742212 (PMC12894019; doi:10.3389/fphar.2026.1742212)
Supplement: Supplementary file 2 [file Table2.docx]

**Supplementary Table S2.** Detailed experimental parameters, controls, and PAINS risk assessment for the natural products listed in Table 2.

| **PCD type** | **Natural products** | **Duration** | **Type of extract** | **Controls** | **Toxic side effects** | **Obstacles to development** | **PAINS risk** | **Evidence level** | **Notes on PAINS assessment** |
| --- | --- | --- | --- | --- | --- | --- | --- | --- | --- |
| Autophagy | Polyphyllin I | 24 h | Pure compound | Untreated cells | Not assessed | Lack of clinical data; Unknown pharmacokinetics; Lack of *in vivo* validation | Low risk (steroidal saponin class, no known PAINS substructures) | Level 3 (verified *in vitro*) |  |
| Autophagy | Carvacrol | In vitro: 48 h; In vivo: 4 w | Pure compound | In vitro: Untreated cells; *In vivo*:  Vehicle control (10% DMSO PBS) | Not assessed | Lack of clinical data; Unknown pharmacokinetics; Small sample size in mouse model (n=4) | Medium risk (monoterpenoid phenol class, contains polyhydroxy/alkoxy benzene motifs associated with PAINS, requiring verification) | Level 2 *(in vivo* + mechanistic exploration) | Potential PAINS liability, *in vivo* evidence mitigates but does not eliminate risk. |
| Autophagy | Saikosaponin A | 24 h | Pure compound | Untreated cells | Reduced cell viability at ≥15 µM (suggests potential cytotoxicity) | Lack of clinical data; Unknown pharmacokinetics; Lack of *in vivo* model validation; Potential low bioavailability | Low risk (triterpenoid saponin class, no known PAINS substructures) | Level 3 (verified *in vitro*) |  |
| Autophagy | Timosaponin AⅢ | 24 h | Pure compound | Untreated cells | Not assessed | Lack of clinical data; Unknown pharmacokinetics; Lack of *in vivo* model validation; Potential low oral bioavailability; Lack of chronic and reproductive toxicity assessment | Low risk (steroidal saponin class, no known PAINS substructures) | Level 4 (preliminary *in vitro*) |  |
| Autophagy | Alpinumisoflavone | 48 h | Pure compound | Vehicle-treated cells | Not assessed | Lack of *in vivo* model validation; Unknown pharmacokinetics; Only in immortalized cell lines (oversimplified, lacks disease microenvironment) | High risk (isoflavone class, contains an α,β-unsaturated carbonyl group (Michael acceptor), a known PAINS feature) | Level 4 (preliminary *in vitro*) | High PAINS risk, results require cautious interpretation |
| Autophagy | Berberine | 2 h | Pure compound | Untreated cells | No significant toxicity at ≤20 µM | Lack of *in vivo* model validation; Unknown pharmacokinetics; Very low oral bioavailability; Bovine cell model limits direct extrapolation to human disease | Medium risk (isoquinoline alkaloid class, contains quaternary ammonium and extended conjugated systems associated with PAINS, requiring verification) | Level 3 (verified *in vitro*) | Potential PAINS liability requires verification in orthogonal assays |
| Autophagy | Quercetin | 4 w | Pure compound | Vehicle control group | Not assessed | Lack of clinical data; Unknown pharmacokinetics | High risk (flavonol class, contains the catechol_A substructure, a known PAINS motif) | Level 2 *(in vivo* + mechanistic exploration) | High PAINS risk, results require cautious interpretation |
| Autophagy | Protopanaxadiol | *In vitro*: 48 h; *In vivo*: 14 d | Pure compound | *In vitro*: DMSO vehicle control, untreated cells, positive control (EsA). *In vivo*: Model control group (injected with an equivalent volume of DMSO vehicle) | Not assessed | Lack of clinical data; Unknown pharmacokinetics; Potential low bioavailability | Low risk (triterpenoid class, no known PAINS substructures) | Level 2 *(in vivo* + mechanistic exploration) |  |
| Autophagy | Gamma oryzanol | 4 w | Extract | EMs-induced group (saline) | Safe at 3, 6 mg/kg | Lack of clinical data; Unknown pharmacokinetics; Studied as a mixture | Medium risk (ferulic acid ester mixture class, contains polyhydroxy cinnamate motifs associated with PAINS, requiring verification) | Level 2 (*in vivo* + mechanistic exploration) | Potential PAINS liability, *in vivo* evidence mitigates but does not eliminate risk. |
| Autophagy | Soy isoflavones | 6 w | Commercial tablets (50 mg isoflavones, 27 mg genistein per tablet) | Saline-treated EMs-induced group | Not assessed | Lack of clinical data; Unknown pharmacokinetics; Long-term safety not assessed; Studied as a mixture | Medium risk (isoflavone class, contains polyhydroxy motifs associated with PAINS, requiring verification) | Level 2 (*in vivo* + mechanistic exploration) | Potential PAINS liability, *in vivo* evidence mitigates but does not eliminate risk. |
| Autophagy | SCM-198 | *In vitro*: 48 h; *In vivo*: 1 w | Purified synthetic compound | *In vitro*: untreated cells. *In vivo*: PBS-treated EMs-induced mice | Not assessed | Lack of clinical data; Unknown pharmacokinetics; Long-term safety not assessed | Medium risk (pyrazolone derivative class, contains redox-active ortho-quinone/phenol motifs associated with PAINS, requiring verification) | Level 2 (*in vivo* + mechanistic exploration) | Potential PAINS liability, *in vivo* evidence mitigates but does not eliminate risk. |
| Autophagy | Paeonol | *In vitro*: 24 h; *In vivo*: 21 d | Pure compound | *In vitro:* Untreated cells, cells transfected with an empty vector.  *In vivo:* Sham surgery group | *In vitro*: Safe at ≤100 µM; *In vivo*: Not assessed | Lack of clinical data; Unknown pharmacokinetics; Low oral bioavailability | Medium risk (phenolic aldehyde class, contains ortho-methoxyphenol motifs associated with PAINS, requiring verification) | Level 2 (*in vivo* + mechanistic exploration) | Potential PAINS liability, *in vivo* evidence mitigates but does not eliminate risk. |
| Ferroptosis | β-elemene | *In vitro*: 48 h; *In vivo*: 21 d | Pure compound | *In vitro*: Untreated cells; erastin as positive control.  *In vivo*: Model group (normal saline); dienogest as positive control | Not assessed | Lack of clinical data; Unknown pharmacokinetics; Potential low oral bioavailability | Low risk (sesquiterpene class, no known PAINS substructures) | Level 2 (*in vivo* + mechanistic exploration) |  |
| Ferroptosis | Resveratrol | *In vitro*: 48 h; *In vivo*: 14 d | Pure compound | *In vitro*: Untreated cells; scramble sequences (mimics-NC, inhibitor-NC) as transfection controls. *In vivo*: Sham surgery group; Model group (solvent) | *In vitro*: Safe at ≤ 200 μM. *In vivo*: Not assessed | Lack of clinical data; Unknown pharmacokinetics; Low oral bioavailability | Medium risk (stilbene class, contains polyhydroxy stilbene motifs associated with PAINS, requiring verification) | Level 2 (*in vivo* + mechanistic exploration) | Potential PAINS liability, *in vivo* evidence mitigates but does not eliminate risk. |
| Ferroptosis | Ginsenoside Rf | *In vitro*: 24 h; *In vivo*: 28 d | Pure compound | *In vitro*: Control group (normal endometrial cells), EMs group (untreated model cells). *In vivo*: Sham surgery group, Model group (normal saline) | Not assessed | Lack of clinical data; Unknown pharmacokinetics; Inconvenient intraperitoneal administration route; Long-term and organ-specific toxicity not assessed | Low risk (triterpenoid saponin class, no known PAINS substructures) | Level 2 (*in vivo* + mechanistic exploration) |  |
| Ferroptosis | Baicalein | 24 h | Pure compound | Untreated THP-1-derived macrophages; DMSO vehicle control | No cytotoxicity was reported at the concentration used (20 µM) | Lack of clinical data; Unknown pharmacokinetics; Lack of *in vivo* model validation; Potential low bioavailability**;** Weak relevance (non-diseased macrophage cell line) | High risk (flavone class, contains the catechol_A substructure, a known PAINS motif) | Level3 (verified *in vitro*) | High PAINS risk, results require cautious interpretation |
| Ferroptosis | Pachymic acid | *In vitro*: 24 h; *In vivo*: 14 d | Pure compound | *In vitro*: Untreated control cells; *In vivo*: Sham-operation group | Not assessed | Lack of clinical data; Unknown pharmacokinetics; Potential low bioavailability | Low risk (triterpenoid acid class, no known PAINS substructures) | Level2 (*in vivo* + mechanistic exploration) |  |
| Ferroptosis | Wogonin | 4 w | Pure compound | Model group | Not assessed | Lack of clinical data; Unknown pharmacokinetics; Potential low bioavailability | Low risk (flavone class, no known PAINS substructures) | Level2 (*in vivo* + mechanistic exploration) |  |
| Pyroptosis | Fisetin | 14 d | Pure compound | Sham group (intraperitoneal PBS injection only, no endometrial tissue transplantation) | No overt toxicity reported in cited rat studies | Lack of clinical data; Unknown pharmacokinetics | High risk (flavonol class, contains the catechol_A substructure, a known PAINS motif) | Level2 (*in vivo* + mechanistic exploration) | High PAINS risk, results require cautious interpretation |
| Pyroptosis | Tetramethylpyrazine | 4 w | Pure compound | Sham surgery group; EMs model group (vehicle control); multiple positive drug control groups (GTN, Vitamin C, Indomethacin) | Not assessed | Lack of clinical data; Unknown pharmacokinetics; Potential low oral bioavailability | Low risk (pyrazine alkaloid class, no known PAINS substructures) | Level 2 (*in vivo* + mechanistic exploration) |  |
| Pyroptosis | CHS-Iva | *In vitro*: 12 h; *In vivo*: 4 w | Pure compound | *In vitro*: Untreated hESCs. *In vivo*: Sham surgery group; EMs model group (Vehicle control) | *In vitro*: Safe at ≤ 50 µM (assessed by CCK-8). *In vivo*: Not assessed | Lack of pharmacokinetic and toxicological data; Lack of clinical data; Potential low oral bioavailability | High risk (chalcone class, contains an α,β-unsaturated ketone (Michael acceptor), a known PAINS feature) | Level 2 (*in vivo* + mechanistic exploration) | High PAINS risk, results require cautious interpretation |
| Pyroptosis | Curcumin | *In vitro*: 24 h; *In vivo*: 4 w | Pure compound | *In vitro*: Untreated cells. *In vivo*: Sham or normal mice (Control); EMs model group (Vehicle control); Dienogest drug control group | Not assessed | Extremely low oral bioavailability; Lack of pharmacokinetic and long-term toxicity data; Lack of clinical data | High risk (diarylheptanoid class, contains β-diketone/enol and Michael acceptor moieties, known PAINS features) | Level 2 (*in vivo* + mechanistic exploration) | High PAINS risk, results require cautious interpretation |
| Pyroptosis | Paeonol | *In vitro*: 48 h; *In vivo*: 4 w | Pure compound | *In vitro*: Untreated mEECs. *In vivo*: Sham surgery group; Model group (Vehicle control); Gestrinone drug control group | Not assessed | Lack of pharmacokinetic and toxicological data; Lack of clinical data | Medium risk (phenolic aldehyde class, contains ortho-methoxyphenol motifs associated with PAINS, requiring verification) | Level 2 (*in vivo* + mechanistic exploration) | Potential PAINS liability, *in vivo* evidence mitigates but does not eliminate risk. |
