# Supplementary material for: Natural products modulate programmed cell death signaling mechanism for treating endometriosis: a review
Source: Front Pharmacol. 2026 Jan 29;17:1742212. doi: 10.3389/fphar.2026.1742212 (PMC12894019; doi:10.3389/fphar.2026.1742212)
Supplement: Supplementary file 3 [file DataSheet1.pdf]

Date of Search: [October 01, 2025]

1 PubMed (91)
1.1 EMs-related terms (62013)
MeSH: Endometriosis
Entry Terms: Endometrioses OR Endometrioma OR Endometriomas OR Endometriosis

Search query: "Endometriosis"[MeSH Terms] OR "Endometrioses"[Title/Abstract] OR "Endometrioma"[Title/Abstract] OR "Endometriomas"[Title/Abstract] OR "Endometriosis"[Title/Abstract] OR "EMs"[Title/Abstract] OR (endometrial stromal cells) OR (Endometriotic Stromal Cells) OR (Endometriotic Cells) **62013**
1.2 Types of PCD (450713)
1.2.1 MeSH: Apoptosis
Entry Terms: Classical Apoptosis OR Apoptosis, Classical OR Classic Apoptosis OR Apoptosis, Classic OR Classic Apoptoses OR Programmed Cell Death, Type I OR Apoptosis, Extrinsic Pathway OR Apoptoses, Extrinsic Pathway OR Extrinsic Pathway Apoptoses OR Extrinsic Pathway Apoptosis OR Apoptosis, Intrinsic Pathway OR Apoptoses, Intrinsic Pathway OR Intrinsic Pathway Apoptoses OR Intrinsic Pathway Apoptosis OR Programmed Cell Death OR Cell Death, Programmed OR Caspase-Dependent Apoptosis OR Apoptosis, Caspase-Dependent OR Caspase Dependent Apoptosis
1.2.2 MeSH: Autophagy
Entry Terms: Autophagocytosis OR Autophagy, Cellular OR Cellular Autophagy OR Lipophagy OR Ribophagy OR Reticulophagy OR ER-Phagy OR ER Phagy OR Nucleophagy
1.2.3 MeSH: Ferroptosis
Entry Terms: Oxytosis
1.2.4 MeSH: Pyroptosis
Entry Terms: Pyroptoses OR Inflammatory Apoptosis OR Apoptoses, Inflammatory OR Apoptosis, Inflammatory OR Inflammatory Apoptoses OR Pyroptotic Cell Death OR Cell Death, Pyroptotic OR Cell Deaths, Pyroptotic OR Death, Pyroptotic Cell OR Deaths, Pyroptotic Cell OR Pyroptotic Cell Deaths OR Caspase-1 Dependent Cell Death OR Caspase 1 Dependent Cell Death
1.2.5 MeSH: Necroptosis
No Entry Terms.
1.2.6 Programmed Cell Death[Title/Abstract] OR PCD[Title/Abstract]

(1.2.1) OR (1.2.2) OR (1.2.3) OR (1.2.4) OR (1.2.5) OR (1.2.6) Search query:
(("Apoptosis"[Mesh]) OR (Classical Apoptosis[Title/Abstract] OR Apoptosis, Classical[Title/Abstract] OR Classic Apoptosis[Title/Abstract] OR Apoptosis, Classic[Title/Abstract] OR Classic Apoptoses[Title/Abstract] OR Programmed Cell Death, Type I[Title/Abstract] OR Apoptosis, Extrinsic Pathway[Title/Abstract] OR Apoptoses, Extrinsic Pathway[Title/Abstract] OR Extrinsic Pathway

Apoptoses[Title/Abstract] OR Extrinsic Pathway Apoptosis[Title/Abstract] OR Apoptosis, Intrinsic Pathway[Title/Abstract] OR Apoptoses, Intrinsic Pathway[Title/Abstract] OR Intrinsic Pathway Apoptoses[Title/Abstract] OR Intrinsic Pathway Apoptosis[Title/Abstract] OR Programmed Cell Death[Title/Abstract] OR Cell Death, Programmed[Title/Abstract] OR Caspase-Dependent Apoptosis[Title/Abstract] OR Apoptosis, Caspase-Dependent[Title/Abstract] OR Caspase Dependent Apoptosis[Title/Abstract])) OR (("Autophagy"[Mesh]) OR (Autophagocytosis[Title/Abstract] OR Autophagy, Cellular[Title/Abstract] OR Cellular Autophagy[Title/Abstract] OR Lipophagy[Title/Abstract] OR Ribophagy[Title/Abstract] OR Reticulophagy[Title/Abstract] OR ER-Phagy[Title/Abstract] OR ER Phagy[Title/Abstract] OR Nucleophagy[Title/Abstract])) OR (("Ferroptosis"[Mesh]) OR (Oxytosis[Title/Abstract])) OR (("Pyroptosis"[Mesh]) OR (Pyroptoses[Title/Abstract] OR Inflammatory Apoptosis[Title/Abstract] OR Apoptoses, Inflammatory[Title/Abstract] OR Apoptosis, Inflammatory[Title/Abstract] OR Inflammatory Apoptoses[Title/Abstract] OR Pyroptotic Cell Death[Title/Abstract] OR Cell Death, Pyroptotic[Title/Abstract] OR Cell Deaths, Pyroptotic[Title/Abstract] OR Death, Pyroptotic Cell[Title/Abstract] OR Deaths, Pyroptotic Cell[Title/Abstract] OR Pyroptotic Cell Deaths[Title/Abstract] OR Caspase-1 Dependent Cell Death[Title/Abstract] OR Caspase 1 Dependent Cell Death[Title/Abstract])) OR ("Necroptosis"[Mesh]) OR (Programmed Cell Death[Title/Abstract]) OR (PCD[Title/Abstract]) **450713**

1.3 Natural products (2706388)

1.3.1 MeSH: Biological Products

Entry Terms: Biologic Product OR Product, Biologic OR Products, Biological OR Biologic Products OR Biological Product OR Product, Biological OR Natural Products OR Natural Product OR Product, Natural OR Biopharmaceuticals OR Biopharmaceutical OR Biological OR Biologic OR Biological Drug OR Drug, Biological OR Biologic Drugs OR Drugs, Biologic OR Biological Drugs OR Drugs, Biological OR Biological Medicines OR Medicines, Biological OR Biologicals OR Biologic Medicines OR Medicines, Biologic OR Biologic Pharmaceuticals OR Pharmaceuticals, Biologic OR Biologics OR Biologic Drug OR Drug, Biologic OR Biological Medicine OR Medicine, Biological

1.3.2 MeSH: Phytochemicals

Entry Terms: Biologically Active Compounds, Plant OR Plant-Derived Compound OR Compound, Plant-Derived OR Plant Derived Compound OR Dietary Phytochemical OR Phytochemical, Dietary OR Plant Bioactive Compound OR Bioactive Compound, Plant OR Compound, Plant Bioactive OR Plant Biologically Active Compound OR Dietary Phytochemicals OR Phytochemicals, Dietary OR Plant Bioactive Compounds OR Bioactive Compounds, Plant OR Compounds, Plant Bioactive OR Plant Biologically Active Compounds OR Plant-Derived Chemical OR Chemical, Plant-Derived OR Plant Derived Chemical OR Bioactive Coumpounds, Plant OR Coumpounds, Plant Bioactive OR Plant Bioactive Coumpounds OR Phytochemical OR Phytonutrient OR Plant-Derived Chemicals OR Chemicals, Plant-Derived OR Plant Derived Chemicals OR Phytonutrients OR Plant-Derived Compounds OR Compounds,

Plant-Derived OR Plant Derived Compounds

1.3.3 MeSH: Plant Extracts

Entry Terms: Extracts, Plant OR Plant Extract OR Extract, Plant OR Herbal Medicines OR Medicines, Herbal

1.3.4 MeSH: Drugs, Chinese Herbal

Entry Terms: Chinese Drugs, Plant OR Chinese Herbal Drugs OR Herbal Drugs, Chinese OR Plant Extracts, Chinese OR Chinese Plant Extracts OR Extracts, Chinese Plant

1.3.5 MeSH: Herbal Medicine

Entry Terms: Medicine, Herbal OR Herbalism OR Hawaiian Herbal Medicine OR Herbal Medicine, Hawaiian OR Medicine, Hawaiian Herbal OR Laau Lapaau OR La au Lapa au OR La'au Lapa'au

1.3.6 MeSH: Plants, Medicinal

Entry Terms: Medicinal Plant OR Plant, Medicinal OR Medicinal Plants OR Medicinal Herbs OR Herb, Medicinal OR Medicinal Herb OR Herbs, Medicinal OR Pharmaceutical Plants OR Pharmaceutical Plant OR Plant, Pharmaceutical OR Plants, Pharmaceutical OR Healing Plants OR Healing Plant OR Plant, Healing OR Plants, Healing

1.3.7 MeSH: Phytotherapy

Entry Terms: Herb Therapy OR Herbal Therapy

(1.3.1) OR (1.3.2) OR (1.3.3) OR (1.3.4) OR (1.3.5) OR (1.3.6) OR (1.3.7) Search query:

((("Biological Products"[Mesh]) OR (Biologic Product[Title/Abstract] OR Product, Biologic[Title/Abstract] OR Products, Biological[Title/Abstract] OR Biologic Products[Title/Abstract] OR Biological Product[Title/Abstract] OR Product, Biological[Title/Abstract] OR Natural Products[Title/Abstract] OR Natural Product[Title/Abstract] OR Product, Natural[Title/Abstract] OR Biopharmaceuticals[Title/Abstract] OR Biopharmaceutical[Title/Abstract] OR Biological[Title/Abstract] OR Biologic[Title/Abstract] OR Biological Drug[Title/Abstract] OR Drug, Biological[Title/Abstract] OR Biologic Drugs[Title/Abstract] OR Drugs, Biologic[Title/Abstract] OR Biological Drugs[Title/Abstract] OR Drugs, Biological[Title/Abstract] OR Biological Medicines[Title/Abstract] OR Medicines, Biological[Title/Abstract] OR Biologicals[Title/Abstract] OR Biologic Medicines[Title/Abstract] OR Medicines, Biologic[Title/Abstract] OR Biologic Pharmaceuticals[Title/Abstract] OR Pharmaceuticals, Biologic[Title/Abstract] OR Biologics[Title/Abstract] OR Biologic Drug[Title/Abstract] OR Drug, Biologic[Title/Abstract] OR Biological Medicine[Title/Abstract] OR Medicine, Biological[Title/Abstract])) OR (("Phytochemicals"[Mesh]) OR (Biologically Active Compounds, Plant[Title/Abstract] OR Plant-Derived Compound[Title/Abstract] OR Compound, Plant-Derived[Title/Abstract] OR Plant Derived Compound[Title/Abstract] OR Dietary Phytochemical[Title/Abstract] OR Phytochemical, Dietary[Title/Abstract] OR Plant Bioactive Compound[Title/Abstract] OR Bioactive Compound, Plant[Title/Abstract]

OR Compound, Plant Bioactive[Title/Abstract] OR Plant Biologically Active Compound[Title/Abstract] OR Dietary Phytochemicals[Title/Abstract] OR Phytochemicals, Dietary[Title/Abstract] OR Plant Bioactive Compounds[Title/Abstract] OR Bioactive Compounds, Plant[Title/Abstract] OR Compounds, Plant Bioactive[Title/Abstract] OR Plant Biologically Active Compounds[Title/Abstract] OR Plant-Derived Chemical[Title/Abstract] OR Chemical, Plant-Derived[Title/Abstract] OR Plant Derived Chemical[Title/Abstract] OR Bioactive Coumpounds, Plant[Title/Abstract] OR Coumpounds, Plant Bioactive[Title/Abstract] OR Plant Bioactive Coumpounds[Title/Abstract] OR Phytochemical[Title/Abstract] OR Phytonutrient[Title/Abstract] OR Plant-Derived Chemicals[Title/Abstract] OR Chemicals, Plant-Derived[Title/Abstract] OR Plant Derived Chemicals[Title/Abstract] OR Phytonutrients[Title/Abstract] OR Plant-Derived Compounds[Title/Abstract] OR Compounds, Plant-Derived[Title/Abstract] OR Plant Derived Compounds[Title/Abstract])) OR (("Plant Extracts"[Mesh]) OR (Extracts, Plant[Title/Abstract] OR Plant Extract[Title/Abstract] OR Extract, Plant[Title/Abstract] OR Herbal Medicines[Title/Abstract] OR Medicines, Herbal[Title/Abstract])) OR (("Drugs, Chinese Herbal"[Mesh]) OR (Chinese Drugs, Plant[Title/Abstract] OR Chinese Herbal Drugs[Title/Abstract] OR Herbal Drugs, Chinese[Title/Abstract] OR Plant Extracts, Chinese[Title/Abstract] OR Chinese Plant Extracts[Title/Abstract] OR Extracts, Chinese Plant[Title/Abstract])) OR (("Herbal Medicine"[Mesh]) OR (Medicine, Herbal[Title/Abstract] OR Herbalism[Title/Abstract] OR Hawaiian Herbal Medicine[Title/Abstract] OR Herbal Medicine, Hawaiian[Title/Abstract] OR Medicine, Hawaiian Herbal[Title/Abstract] OR Laau Lapaau[Title/Abstract] OR La au Lapa au[Title/Abstract] OR La'au Lapa'au[Title/Abstract])) OR (("Plants, Medicinal"[Mesh]) AND (Medicinal Plant[Title/Abstract] OR Plant, Medicinal[Title/Abstract] OR Medicinal Plants[Title/Abstract] OR Medicinal Herbs[Title/Abstract] OR Herb, Medicinal[Title/Abstract] OR Medicinal Herb[Title/Abstract] OR Herbs, Medicinal[Title/Abstract] OR Pharmaceutical Plants[Title/Abstract] OR Pharmaceutical Plant[Title/Abstract] OR Plant, Pharmaceutical[Title/Abstract] OR Plants, Pharmaceutical[Title/Abstract] OR Healing Plants[Title/Abstract] OR Healing Plant[Title/Abstract] OR Plant, Healing[Title/Abstract] OR Plants, Healing[Title/Abstract])) OR (("Phytotherapy"[Mesh]) OR (Herb Therapy[Title/Abstract] OR Herbal Therapy[Title/Abstract]))) OR ((chinese medicine)OR(Chinese herbal monomer)OR(Chinese herbal extract) OR(Chinese herbal medicine) OR(Chinese herbal monomers)OR(Traditional Chinese medicine) OR(medicinal plants) OR(active ingredients) OR(herbal formula) OR(natural compounds)OR(natural products) OR(phytochemicals) OR(herbal extracts)OR(plant-derived)) **2706388**

1.4 Mechanistic Research (2354639)

MeSH: Signal Transduction

Entry Terms: Signal Transductions OR Transduction, Signal OR Transductions, Signal OR Cell Signaling OR Signal Transduction Systems OR Signal Transduction System OR System, Signal Transduction OR Systems, Signal Transduction OR Receptor-

Mediated Signal Transduction OR Receptor-Mediated Signal Transductions OR Signal Transduction, Receptor-Mediated OR Signal Transductions, Receptor-Mediated OR Receptor Mediated Signal Transduction OR Signal Pathways OR Pathway, Signal OR Pathways, Signal OR Signal Pathway OR Signal Transduction Pathways OR Pathway, Signal Transduction OR Pathways, Signal Transduction OR Signal Transduction Pathway

Search query: (("Signal Transduction"[Mesh]) OR (Signal Transductions[Title/Abstract] OR Transduction, Signal[Title/Abstract] OR Transductions, Signal[Title/Abstract] OR Cell Signaling[Title/Abstract] OR Signal Transduction Systems[Title/Abstract] OR Signal Transduction System[Title/Abstract] OR System, Signal Transduction[Title/Abstract] OR Systems, Signal Transduction[Title/Abstract] OR Receptor-Mediated Signal Transduction[Title/Abstract] OR Receptor-Mediated Signal Transductions[Title/Abstract] OR Signal Transduction, Receptor-Mediated[Title/Abstract] OR Signal Transductions, Receptor-Mediated[Title/Abstract] OR Receptor Mediated Signal Transduction[Title/Abstract] OR Signal Pathways[Title/Abstract] OR Pathway, Signal[Title/Abstract] OR Pathways, Signal[Title/Abstract] OR Signal Pathway[Title/Abstract] OR Signal Transduction Pathways[Title/Abstract] OR Pathway, Signal Transduction[Title/Abstract] OR Pathways, Signal Transduction[Title/Abstract] OR Signal Transduction Pathway[Title/Abstract])) OR (mechanism[Title/Abstract]) **2354639**

(1.1) AND (1.2) AND (1.3) AND (1.4) Search query:
((((("Endometriosis"[MeSH Terms] OR "Endometrioses"[Title/Abstract] OR "Endometrioma"[Title/Abstract] OR "Endometriomas"[Title/Abstract] OR "Endometriosis"[Title/Abstract] OR "EMs"[Title/Abstract]OR(endometrial stromal cells)OR(Endometriotic Stromal Cells)OR(Endometriotic Cells))) AND ((("Apoptosis"[Mesh]) OR (Classical Apoptosis[Title/Abstract] OR Apoptosis, Classical[Title/Abstract] OR Classic Apoptosis[Title/Abstract] OR Apoptosis, Classic[Title/Abstract] OR Classic Apoptoses[Title/Abstract] OR Programmed Cell Death, Type I[Title/Abstract] OR Apoptosis, Extrinsic Pathway[Title/Abstract] OR Apoptoses, Extrinsic Pathway[Title/Abstract] OR Extrinsic Pathway Apoptoses[Title/Abstract] OR Extrinsic Pathway Apoptosis[Title/Abstract] OR Apoptosis, Intrinsic Pathway[Title/Abstract] OR Apoptoses, Intrinsic Pathway[Title/Abstract] OR Intrinsic Pathway Apoptoses[Title/Abstract] OR Intrinsic Pathway Apoptosis[Title/Abstract] OR Programmed Cell Death[Title/Abstract] OR Cell Death, Programmed[Title/Abstract] OR Caspase-Dependent Apoptosis[Title/Abstract] OR Apoptosis, Caspase-Dependent[Title/Abstract] OR Caspase Dependent Apoptosis[Title/Abstract])) OR (("Autophagy"[Mesh]) OR (Autophagocytosis[Title/Abstract] OR Autophagy, Cellular[Title/Abstract] OR Cellular Autophagy[Title/Abstract] OR Lipophagy[Title/Abstract] OR Ribophagy[Title/Abstract] OR Reticulophagy[Title/Abstract] OR ER-Phagy[Title/Abstract] OR ER Phagy[Title/Abstract] OR Nucleophagy[Title/Abstract]))

OR (("Ferroptosis"[Mesh]) OR (Oxytosis[Title/Abstract])) OR (("Pyroptosis"[Mesh]) OR (Pyroptoses[Title/Abstract] OR Inflammatory Apoptosis[Title/Abstract] OR Apoptoses, Inflammatory[Title/Abstract] OR Apoptosis, Inflammatory[Title/Abstract] OR Inflammatory Apoptoses[Title/Abstract] OR Pyroptotic Cell Death[Title/Abstract] OR Cell Death, Pyroptotic[Title/Abstract] OR Cell Deaths, Pyroptotic[Title/Abstract] OR Death, Pyroptotic Cell[Title/Abstract] OR Deaths, Pyroptotic Cell[Title/Abstract] OR Pyroptotic Cell Deaths[Title/Abstract] OR Caspase-1 Dependent Cell Death[Title/Abstract] OR Caspase 1 Dependent Cell Death[Title/Abstract])) OR ("Necroptosis"[Mesh]) OR (Programmed Cell Death[Title/Abstract]) OR (PCD[Title/Abstract]))) AND (((("Biological Products"[Mesh]) OR (Biologic Product[Title/Abstract] OR Product, Biologic[Title/Abstract] OR Products, Biological[Title/Abstract] OR Biologic Products[Title/Abstract] OR Biological Product[Title/Abstract] OR Product, Biological[Title/Abstract] OR Natural Products[Title/Abstract] OR Natural Product[Title/Abstract] OR Product, Natural[Title/Abstract] OR Biopharmaceuticals[Title/Abstract] OR Biopharmaceutical[Title/Abstract] OR Biological[Title/Abstract] OR Biologic[Title/Abstract] OR Biological Drug[Title/Abstract] OR Drug, Biological[Title/Abstract] OR Biologic Drugs[Title/Abstract] OR Drugs, Biologic[Title/Abstract] OR Biological Drugs[Title/Abstract] OR Drugs, Biological[Title/Abstract] OR Biological Medicines[Title/Abstract] OR Medicines, Biological[Title/Abstract] OR Biologicals[Title/Abstract] OR Biologic Medicines[Title/Abstract] OR Medicines, Biologic[Title/Abstract] OR Biologic Pharmaceuticals[Title/Abstract] OR Pharmaceuticals, Biologic[Title/Abstract] OR Biologics[Title/Abstract] OR Biologic Drug[Title/Abstract] OR Drug, Biologic[Title/Abstract] OR Biological Medicine[Title/Abstract] OR Medicine, Biological[Title/Abstract])) OR (("Phytochemicals"[Mesh]) OR (Biologically Active Compounds, Plant[Title/Abstract] OR Plant-Derived Compound[Title/Abstract] OR Compound, Plant-Derived[Title/Abstract] OR Plant Derived Compound[Title/Abstract] OR Dietary Phytochemical[Title/Abstract] OR Phytochemical, Dietary[Title/Abstract] OR Plant Bioactive Compound[Title/Abstract] OR Bioactive Compound, Plant[Title/Abstract] OR Compound, Plant Bioactive[Title/Abstract] OR Plant Biologically Active Compound[Title/Abstract] OR Dietary Phytochemicals[Title/Abstract] OR Phytochemicals, Dietary[Title/Abstract] OR Plant Bioactive Compounds[Title/Abstract] OR Bioactive Compounds, Plant[Title/Abstract] OR Compounds, Plant Bioactive[Title/Abstract] OR Plant Biologically Active Compounds[Title/Abstract] OR Plant-Derived Chemical[Title/Abstract] OR Chemical, Plant-Derived[Title/Abstract] OR Plant Derived Chemical[Title/Abstract] OR Bioactive Coumpounds, Plant[Title/Abstract] OR Coumpounds, Plant Bioactive[Title/Abstract] OR Plant Bioactive Coumpounds[Title/Abstract] OR Phytochemical[Title/Abstract] OR Phytonutrient[Title/Abstract] OR Plant-Derived Chemicals[Title/Abstract] OR Chemicals, Plant-Derived[Title/Abstract] OR Plant Derived Chemicals[Title/Abstract] OR Phytonutrients[Title/Abstract] OR Plant-Derived Compounds[Title/Abstract] OR Compounds, Plant-Derived[Title/Abstract] OR Plant Derived Compounds[Title/Abstract])) OR (("Plant Extracts"[Mesh]) OR

(Extracts, Plant[Title/Abstract] OR Plant Extract[Title/Abstract] OR Extract, Plant[Title/Abstract] OR Herbal Medicines[Title/Abstract] OR Medicines, Herbal[Title/Abstract])) OR (("Drugs, Chinese Herbal"[Mesh]) OR (Chinese Drugs, Plant[Title/Abstract] OR Chinese Herbal Drugs[Title/Abstract] OR Herbal Drugs, Chinese[Title/Abstract] OR Plant Extracts, Chinese[Title/Abstract] OR Chinese Plant Extracts[Title/Abstract] OR Extracts, Chinese Plant[Title/Abstract])) OR (("Herbal Medicine"[Mesh]) OR (Medicine, Herbal[Title/Abstract] OR Herbalism[Title/Abstract] OR Hawaiian Herbal Medicine[Title/Abstract] OR Herbal Medicine, Hawaiian[Title/Abstract] OR Medicine, Hawaiian Herbal[Title/Abstract] OR Laau Lapaau[Title/Abstract] OR La au Lapa au[Title/Abstract] OR La'au Lapa'au[Title/Abstract])) OR (("Plants, Medicinal"[Mesh]) AND (Medicinal Plant[Title/Abstract] OR Plant, Medicinal[Title/Abstract] OR Medicinal Plants[Title/Abstract] OR Medicinal Herbs[Title/Abstract] OR Herb, Medicinal[Title/Abstract] OR Medicinal Herb[Title/Abstract] OR Herbs, Medicinal[Title/Abstract] OR Pharmaceutical Plants[Title/Abstract] OR Pharmaceutical Plant[Title/Abstract] OR Plant, Pharmaceutical[Title/Abstract] OR Plants, Pharmaceutical[Title/Abstract] OR Healing Plants[Title/Abstract] OR Healing Plant[Title/Abstract] OR Plant, Healing[Title/Abstract] OR Plants, Healing[Title/Abstract])) OR (("Phytotherapy"[Mesh]) OR (Herb Therapy[Title/Abstract] OR Herbal Therapy[Title/Abstract]))) OR ((chinese medicine)OR(Chinese herbal monomer)OR(Chinese herbal extract) OR(Chinese herbal medicine) OR(Chinese herbal monomers)OR(Traditional Chinese medicine) OR(medicinal plants) OR(active ingredients) OR(herbal formula) OR(herbal extracts) OR(natural compounds)OR(natural products) OR(phytochemicals) OR(plant-derived)))) AND ((("Signal Transduction"[Mesh]) OR (Signal Transductions[Title/Abstract] OR Transduction, Signal[Title/Abstract] OR Transductions, Signal[Title/Abstract] OR Cell Signaling[Title/Abstract] OR Signal Transduction Systems[Title/Abstract] OR Signal Transduction System[Title/Abstract] OR System, Signal Transduction[Title/Abstract] OR Systems, Signal Transduction[Title/Abstract] OR Receptor-Mediated Signal Transduction[Title/Abstract] OR Receptor-Mediated Signal Transductions[Title/Abstract] OR Signal Transduction, Receptor-Mediated[Title/Abstract] OR Signal Transductions, Receptor-Mediated[Title/Abstract] OR Receptor Mediated Signal Transduction[Title/Abstract] OR Signal Pathways[Title/Abstract] OR Pathway, Signal[Title/Abstract] OR Pathways, Signal[Title/Abstract] OR Signal Pathway[Title/Abstract] OR Signal Transduction Pathways[Title/Abstract] OR Pathway, Signal Transduction[Title/Abstract] OR Pathways, Signal Transduction[Title/Abstract] OR Signal Transduction Pathway[Title/Abstract])) OR (mechanism[Title/Abstract]))

Search time: January 01, 2015--October 01, 2025 91

Records Retrieved: [91]

2 Web of Science (125)

TS=(((endometrios* OR endometrioma* OR "endometrial stromal cells" OR

"endometriotic stromal cells" OR "endometriotic cells") AND ((apoptosis OR "classical apoptosis" OR "classic apoptosis" OR "programmed cell death" OR "caspase-dependent apoptosis" OR "extrinsic pathway" OR "intrinsic pathway") OR (autophagy OR autophagocytosis OR lipophagy OR ribophagy OR reticulophagy OR er-phagy OR nucleophagy) OR (ferroptosis OR oxytosis) OR (pyroptosis OR "inflammatory apoptosis" OR "pyroptotic cell death" OR "caspase-1 dependent cell death") OR (necroptosis) OR ("programmed cell death" OR PCD)) AND (("biological product*" OR "biologic product*" OR "natural product*" OR biopharmaceutical* OR biologic* OR "biological drug*" OR "biological medicine*") OR (phytochemical* OR "plant-derived compound*" OR "plant bioactive compound*" OR "dietary phytochemical*" OR phytonutrient*) OR ("plant extract*" OR "herbal medicine*") OR ("chinese herbal drug*" OR "chinese plant extract*") OR ("herbal medicine" OR herbalism) OR ("medicinal plant*" OR "medicinal herb*" OR "pharmaceutical plant*") OR (phytotherapy OR "herb therapy" OR "herbal therapy") OR ("chinese medicine" OR "chinese herbal monomer*" OR "chinese herbal extract" OR "traditional chinese medicine" OR "active ingredient*" OR "herbal formula" OR "herbal extract*" OR "natural compound*" OR "plant-derived")) AND (("signal transduction" OR "signal pathway*" OR "cell signaling" OR "receptor-mediated signal transduction") OR mechanism)))

Search time: January 01, 2015--October 01, 2025

Records Retrieved: **[125]**

3 CNKI (626)

(SU='子宫内膜异位症' OR SU='异位症') AND (FT='凋亡' OR FT='自噬' OR FT='铁死亡' OR FT='焦亡' OR FT='坏死性凋亡' OR FT='程序性细胞死亡') AND (FT='天然产物' OR FT='中药' OR FT='中药单体'　OR FT='中药提取物' OR FT='植物化学物' OR FT='植物提取物') AND FT='信号通路'

Search time: January 01, 2015--October 01, 2025

Records Retrieved: **[626]**
